# An LED-based multi-sample absorbance spectrophotometer for chemistry and biochemistry

**DOI:** 10.1016/j.ohx.2025.e00690

**Published:** 2025-08-21

**Authors:** David P. Goldenberg

**Affiliations:** School of Biological Sciences, University of Utah, Salt Lake City, UT, United States of America

**Keywords:** Spectrophotometry, Light-emitting diode, Arduino, Education

## Abstract

The instrument described in this article measures the absorbance of visible light (with wavelengths in the range of approximately 400 to 650 nm) by liquid samples, a method widely used for determining solute concentrations. To minimize the cost of the instrument, interchangeable light-emitting diodes (LEDs) are used as light sources. Transmitted light is detected using a photodiode device and the signals are processed using an Arduino microprocessor board. Measured absorbances are displayed on an LCD panel and can be transferred to another device via a USB interface. The instrument has measuring positions for up to six samples, each with a separate LED and detector, making it particularly well suited for parallel kinetic experiments with multiple samples. Because the spectrophotometer was designed with undergraduate laboratory courses in mind, it has a modular construction that allows for easy assembly and disassembly, so that students can be given an opportunity to assemble the instrument themselves. The device has a power requirement of only 0.4 W from a 5 V USB supply, making it practical for field studies or other applications where access to electric power is limited.

## Graphical abstract

**Figure:**
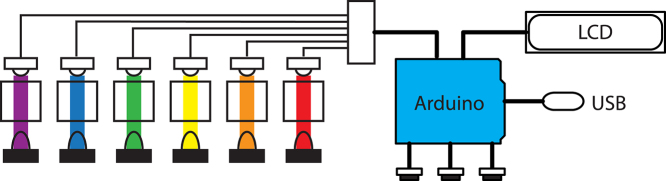

## Specifications table

**Table:** 

Hardware name	LED absorbance spectrophotometer
Subject area	Chemistry and Biochemistry
Hardware type	Laboratory instrumentation
Open source license	CERN Open Hardware License
Cost of hardware	$1500
Source file repository	https://osf.io/qs7r9/

## 1 Hardware in context

Absorption spectrophotometry, based on visible or ultraviolet (UV) light, is a widely used technique in chemistry, biochemistry and other fields, providing a simple and direct means of measuring the concentrations of solutes in liquid samples. The instrumentation for these measurements is conceptually simple and consists of a light source, a sample holder, a photodetector and electronics to convert the signal from the detector to an appropriate output [1]^1^.

Commercially available spectrophotometers range in cost from about $1500 to $20,000, with the less expensive instruments limited to the visible light spectrum, and rather wide spectral width (*e.g.,* 20 nm) and the more expensive ones having additional capabilities for UV measurements and handling multiple samples. Although the cost of a spectrophotometer is not usually prohibitive for a research or diagnostic laboratory in developed nations, the cost can limit the application of the method in educational contexts, such as high school or undergraduate teaching laboratories, and in less wealthy regions. In addition, most conventional instruments are relatively large and delicate, and require AC power sources, thus limiting their portability.

**Fig. 1:**
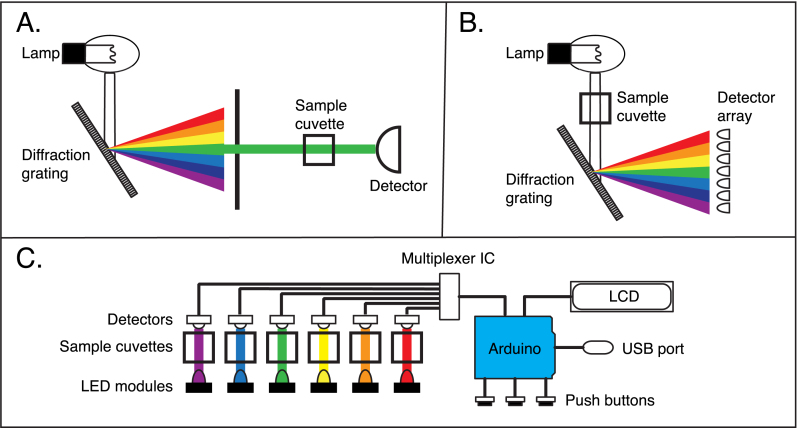
Diagrams illustrating the overall designs of conventional absorbance spectrophotometers (A and B) and the LED spectrophotometer described in this paper (C). Panel A shows the most common configuration of UV–visible spectrophotometers, in which a diffraction grating is used to select light with a narrow distribution of wavelengths, which is then directed to the sample cuvette. In the design illustrated in panel B, broad-spectrum light passes through the sample and is then separated by a diffraction grating. In this design, an array of detectors is used to measure simultaneously the intensities of transmitted light with multiple wavelengths. The LED spectrophotometer (C) eliminates the need for a diffraction grating by using interchangeable LEDs as light sources. In the design described here, there are six sample positions, each with its own LED and photosensor. The signals from the sensors are converted to absorbance measurements by an Arduino microprocessor board and displayed on an LCD panel. An external device, connected to the USB port of the Arduino, can also be used to control the instrument and record data.

The most expensive, complex and delicate component of most spectrophotometers is a monochromator, which is used to provide narrow-bandwidth light from a wide-band source, typically a tungsten, deuterium or xenon lamp [1]. The monochromators in good-quality spectrophotometers are based on diffraction gratings and produce bandwidths as narrow as about 1 nm, and they are typically adjustable from 200 to 650 nm. In these instruments, the monochromator is placed between the light source and the sample (Fig. 1A). Because only a small fraction of the light from the source is used in a single measurement, the energy efficiency of these instruments is quite low. In an alternative configuration (Fig. 1B), wide-band light is directed through the sample and then separated using a diffraction grating and detected with an array detector, so that light from the full spectrum can be recorded simultaneously. Both of these arrangement offer a high degree of precision and flexibility, but both are rather expensive and delicate.

As a means of producing simpler and less expensive instruments, several groups have described the use of light-emitting diodes (LEDs) as light sources [2], [3], [4], [5], [6], [7], [8], [9], [10], [11], [12], [13], [14], [15], [16], [17], [18], [19], [20], [21], [22], [23], [24]. Although LEDs are not monochromatic sources, with typical band widths of approximately 15–40 nm (full width at half maximum, FWHM), the absorption spectra of most compounds are similarly wide, allowing LEDs to be used without filtration for many applications. Many of the initial applications of LEDs for spectrophotometry involved monitoring the compositions of flowing solutions, such as the eluents of chromatography columns, an application where measurements at a single wavelength are often adequate [2], [3], [4]. Other instruments have been designed for fixed samples, with holders for cuvettes that typically hold 1–3 mL of solution, [5], [6], [9], [11], [12], [16], [21], [23], and some have included provisions for multiple LEDs to allow measurements at different wavelengths [3], [5], [6]. Other instruments have been designed for specific applications, such as detecting glucose [18], nitrites [22] or pesticides [11], [23].

Several publications have described the construction of LED spectrophotometers specifically for educational and public-science applications [7], [8], [10], [12], [13], [14], [15], [16], [17], [19], [20], [24], many of which have been reviewed by Kovarik et al. [25]. In addition to their low cost, LED-based instruments can be designed to offer students the opportunity to assemble the instrument themselves. This hands-on experience encourages students to think about the inner design and workings of scientific instruments, rather than treating them as black boxes. This approach also allows the introduction of interdisciplinary content, for instance from physics, electronics and computer programming when a programmable microprocessor is included in the design.

Of the instruments intended for education, many are designed to facilitate simplicity of assembly and low cost, using inexpensive construction materials (such as LEGO® blocks and repurposed containers) and a multimeter to measure the output from a photodiode [7], [8], [10], [15], [20]. These designs are ideal for demonstrating the principles of spectrophotometry, but their construction tends to be rather fragile, and the electrical readings must be converted to absorbance values, thus limiting their application for routine measurements. Some more complex designs utilize 3d-printed components for more robust construction [17], [19], [24], and some incorporate electronics to convert the electrical signal to an absorbance reading [12], [15], [16].

Like most other LED-based instruments intended for educational use, the device described here is designed so that it can be assembled by students, but it extends these designs in important ways. In particular, the instrument can make measurements of up to six samples in parallel, and it can be used either on its own, using push buttons for control and an LCD panel for data output, or can be connected to a computer, via a USB port, for automatic control and data acquisition. The instrument features a modular design and robust construction, so that it can be used throughout a course as a reliable instrument for a variety of experiments, not simply as a demonstration of the principles of spectrophotometry.

Some features of the instrument also make it suitable for applications beyond a classroom setting, such as use in field classes or research sites. In addition to being compact, the device requires very little power (about 0.4 W at 5 V), so that it can be powered from a laptop computer or battery back. And, the instrument can be pre-configured with LEDs with one or more suitable wavelengths, thus minimizing any adjustments in the field.

## 2 Hardware description

As diagrammed in Fig. 1C, the instrument is, in essence, six individual spectrophotometers, with outputs multiplexed to a single Arduino (or compatible) microprocessor board for data processing and output. For each cuvette position, there is a separate photodetector and LED, with the individual LEDs mounted on easily interchangeable modules. The six cuvette positions can thus be configured with LEDs with different peak wavelengths, providing a limited degree of spectral resolution, or they can all be configured with identical LEDs. The latter configuration is particularly useful for kinetic experiments, in which time-dependent changes in concentration in multiple samples can be measured simultaneously. In most conventional spectrophotometers equipped to measure multiple samples, the individual cuvettes are mounted in a moveable holder, and the cuvettes are moved sequentially into a single measurement position. The very low costs of the light sources and detectors used in the LED spectrophotometer allow for a much simpler and rugged mechanical arrangement.

Two important design goals for the instrument were (1) to make it easy for students to assemble themselves and (2) for the construction to be robust enough for repeated assembly and disassembly, as well as for use in a wide range of conditions. The features incorporated to meet these goals include the use of printed circuit boards (PCBs) for most of the circuitry, the use of metal helical threaded inserts for most of the threaded holes in the plastic components and the use of polarized push-on connectors on the PCBs and cables, to minimize possible wiring errors.

In an educational setting it is expected that all of the major components – including circuit boards, cables, CNC-machined parts and the enclosure – would be constructed and presented to the students as a kit, leaving to them the relatively simple final assembly. At the end of one school term, the device can be easily disassembled and then used again in another term. Depending on the amount of class time to be devoted the project, some parts of the assembly, such as the cuvette block and control panel, could be pre-assembled for the students.

**Fig. 2:**
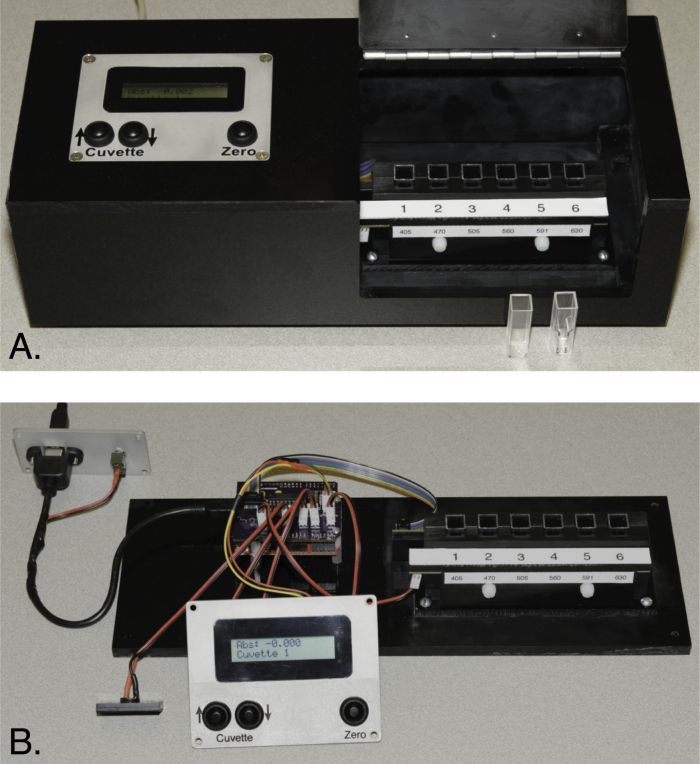
The fully assembled LED spectrophotometer (A) and the instrument with the enclosure removed (B). The instrument is divided into two major compartments, with the electronics on the left, and the cuvette holders, photosensors and LEDs on the right. (The partition separating the compartments is not shown in panel B, to allow the components on both sides to be seen more clearly.) In addition to the components mounted to the instrument base, three assemblies are mounted on the upper enclosure and are shown in panel B: A rear panel with a USB port and power switch (upper left), a small speaker element for alert tones (lower left) and a top panel with the LCD display and control buttons. In both photographs, the instrument is configured with LEDs with different emission wavelengths in the six positions. Examples of plastic cuvettes for 3 mL (left) and 1 mL (right) samples are shown in front of the instrument in panel A.

Photographs of the fully assembled instrument are shown in Fig. 2, and details of the design are described below.

### 2.1 Electronic design

A key component used in the design is the TSL235R light-to-frequency converter (Texas Advanced Optoelectronic Solutions, TAOS). This device combines a photodiode and an integrated circuit that converts the analog output of the photodiode to the frequency of a square-wave output signal. The frequency is directly proportional to light intensity over nearly six orders of magnitude. The frequency of the output is then determined on the Arduino, using the FreqCount software package. Because only a single Arduino pin can be configured for frequency measurement with this package, a multiplexer chip (4HC4051) is used to select among the signals from the six sensors, using digital outputs from the Arduino to control the multiplexer. To select the sample for measurement, the Arduino is controlled, in turn, by either two push buttons on the display panel, or by an external computer connected via the USB port.

Output of the measurement is expressed in dimensionless absorbance units, defined as:

(1) $$A=\log\frac{I_{0}}{I}$$

where $I_{0}$ is the initial light intensity, before passing through the cuvette, and I is the intensity after passing through the cuvette. The usual practice for this type of measurement is to set the absorbance reading to zero when a “blank” solution, lacking only the compound of interest, is placed in the instrument. In the LED spectrophotometer, a push button on the display panel (or an external command) directs the Arduino to record a reference frequency, $f_{r}$, for the blank sample, and the absorbance values are calculated from the frequency, f, measured for a sample as:

(2) $$A=\log\frac{f_{r}}{f}$$

This calculation is performed by the Arduino and the result is displayed on a two-line LCD panel. The LCD panel is a model that incorporates a separate microprocessor that enables serial communication with the Arduino, thus minimizing the number of connections between the Arduino and display. The output can also be transferred to an external device via serial communication over the USB port on the Arduino board. The USB port is also used as the 5 V power input for the instrument.

### 2.2 Mechanical design

**Fig. 3:**
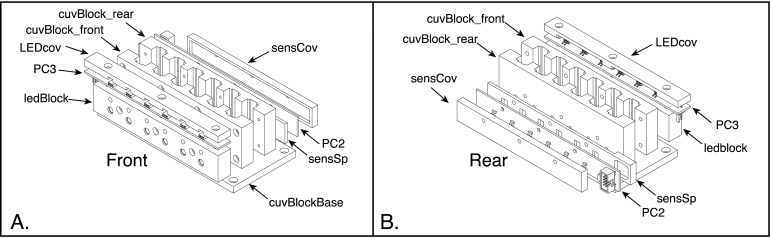
Front (A) and rear (B) exploded views of the core spectrophotometer assembly. The assembly consists of six pieces machined from ACP plastic and 3 printed circuit boards (PC2 and PC3). The part labels correspond to the designators in the Bill of Materials and the design-file names listed in Sections 3.2.1–3.2.4.

The core of the mechanical design is an assembly that holds the sample cuvettes, LEDs and photosensors in careful alignment. As diagrammed in Fig. 3, this assembly is composed of six parts machined from acetal-copolymer (ACP) plastic, along with two PCBs. One of the circuit boards (PC2) holds the TSL235R photodetectors, and the other (PC3) holds surface-mount female connectors used to connect the LED modules to their power source. The assembly is held together with hex-head cap screws.

**Fig. 4:**
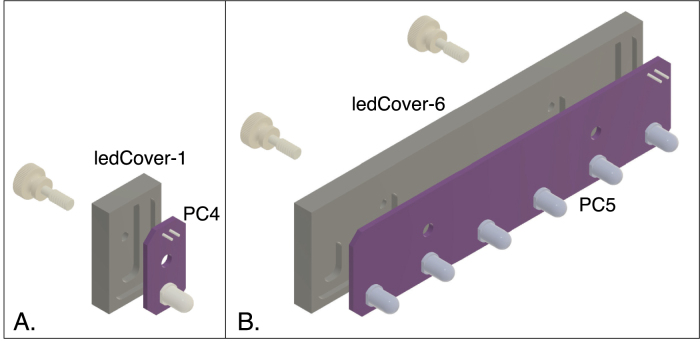
LED modules for a single LED (A) and six LEDs (B). The covers are machined from phenolic resin laminate and glued to the circuit boards (PC4 and PC5). The modules are attached to the LED block of the core assembly using nylon thumbscrews. The part labels correspond to the designators in the Bill of Materials and the design-file names listed in Sections 3.2.1–3.2.4.

Two types of LED modules have been designed, one that holds a single LED and another that holds six. Each module is composed of a circuit board and a cover machined from phenolic resin laminate (Fig. 4). The LEDs are mounted on the circuit board, along with surface-mount voltage-dropping resistors and connecting pins. Phenolic resin is used for the covers, rather than ACP, because it is more easily glued to the circuit boards. These modules are mounted onto the core assembly with thumb screws, and the connecting pins mate with the female connectors on PC3.

The core assembly is mounted on a black acrylic plastic base, along with the Arduino circuit board and a custom board (PC1) that mates with the Arduino connectors and provides the connections to the other electronic components. (A mating board of this type is referred to as a shield in the Arduino community).

**Fig. 5:**
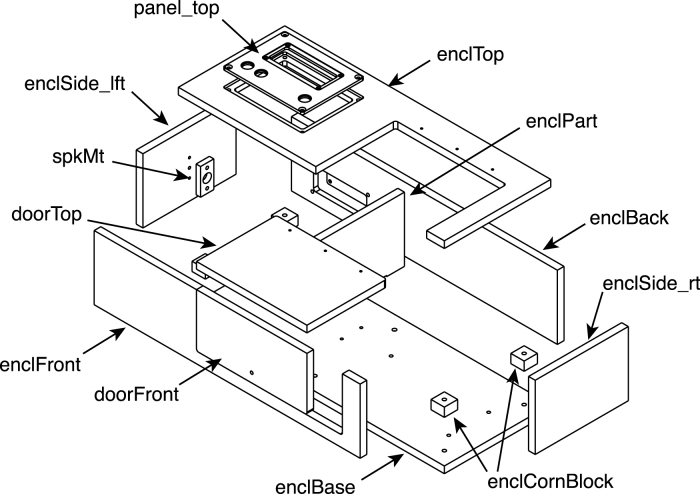
Exploded view of the instrument enclosure. All of the parts are machined from black acrylic plastic sheet, except for the panels at the top and rear, which are machined from aluminum sheet. The rear panel is largely hidden from view in the drawing and is not labeled. The part labels correspond to the designators in the Bill of Materials and the design-file names listed in Sections 3.2.1–3.2.4.

The design of the enclosure, which is made of black acrylic sheet, is illustrated in Fig. 5. The LCD panel is mounted to an aluminum plate in the top panel of the enclosure, along with three control buttons. A second aluminum plate in the rear panel is used to mount a power switch and a female USB connector.

## 3 Design files

### 3.1 Design files summary

**Table:** 

Design filename	File type	Open source license	Location of the file
3d-step.zip	CAD files	CERN OHL	https://osf.io/qs7r9/
PCB.zip	PCB files	CERN OHL	https://osf.io/qs7r9/
LEDspec_arduino.zip	Arduino sketch files	BSD	https://osf.io/qs7r9/
labels.zip	Graphics files	BSD	https://osf.io/qs7r9/

### 3.2 Design-file descriptions

#### 3.2.1 STEP-format 3-d CAD files

The ZIP archive file, 3d-step.zip, contains the following CAD files in STEP format:

• cuvBlock_front.stp: LED side of the cuvette block.
• cuvBlock_rear.stp: Detector side of the cuvette block.
• cuvBlockBase.stp: Base of the cuvette block assembly.
• doorFront.stp: Front part of the door.
• doorTop.stp: Top part of the door.
• enclBack.stp: Enclosure back panel.
• enclBase.stp: Enclosure bottom panel.
• enclCornBlock.stp: Corner blocks of the enclosure, for mounting the enclosure bottom.
• enclFront.stp: Front panel of the enclosure.
• enclPart.stp: Enclosure partition.
• enclSide_lft.stp: Left panel of the enclosure.
• enclSide_rt.stp: Right panel of the enclosure.
• enclTop.stp: Top panel of the enclosure.
• ledBlock.stp: LED block.
• ledConnCover.stp: LED connector cover.
• ledConnJig.stp: Jig used to aid assembly of the led-connector PCB (PC3)
• ledCover-1.stp: Single-LED board cover.
• ledCover-6.stp: 6-LED board cover.
• ledHolderJig.stp: Jig used to aid assembly of the LED module PCBs (PC4 and PC5)
• panel_back.stp: Rear plate for mounting USB connector and power switch.
• panel_top.stp: Top panel, for mounting the LCD panel and control switches.
• sensCov.stp: Sensor-board cover.
• sensSp.stp: Spacer between the cuvette block and the sensor PCB board.
• spkrMt.stp: Mounting bracket for the mini speaker.

#### 3.2.2 PCB design files

The ZIP archive file, PCB.zip, contain native files from Osmond PCB, the MacOS program used to design the boards, and gerber-format files that can be used directly by most PCB manufacturers. The gerber files have also been made available through OSHPARK and hyperlinks to the corresponding web pages are provided in the Bill of Materials. Files are provided for the following PCBs:

• PC1: Arduino shield.
• PC2: Sensor board.
• PC3: LED connector board.
• PC4: Single-LED holder board.
• PC5: LED holder board for six LEDs.

#### 3.2.3 Arduino sketch files

The ZIP archive file, LEDspec_arduino.zip, contains the following Arduino sketch files:

• LEDspec.ino: Sketch for operation of the spectrophotometer
• serLCDsetup.ino: Sketch for configuration of the LCD display

#### 3.2.4 Label files

The ZIP archive file, labels.zip, contains the following graphics files in EPS format:

• cuvPosLabels.eps: Labels to be placed on the top surface of the LED connector cover, to identify the cuvette positions.
• LEDlabels.eps: Label templates for attaching to the LED covers, to identify the LEDs by their wavelength maxima.
• topPlateLabel.eps: Label to apply to the aluminum top panel, to identify the control buttons.

## 4 Bill of materials

The bill of materials is provided as a spreadsheet, ledSpec_bom.ods, which can be downloaded from https://osf.io/qs7r9/.

## 5 Build instructions

### 5.1 General considerations

In most contexts, the most expensive and time-consuming aspect of the construction is likely to be the fabrication of the plastic (ACP), composite (phenolic resin) and metal (aluminum) components of the spectrophotometer. For the instruments that have been assembled so far, these components were machined from sheet stock using a CNC (computer numerical control) milling machine at the University of Utah School of Medicine machine shop, at a cost of about $1100 per unit (including labor and materials). CAD files, in the STEP format, are provided for all of the machined parts. STEP files can be opened in most computer-aided design (CAD) programs for further editing and can be used to create files in the formats required for CNC machines (usually Gcode).

Nearly all of the electronic components are mounted on PCBs, including the Arduino board, an LCD board with onboard serial interface and controller, and five custom boards described further below. Design files for the custom PCBs are provided in Gerber format and in the format for Osmond PCB, the Macintosh program used to design the boards. The Gerber files have also been submitted to OSHPARK (https://oshpark.com/), a commercial manufacturer of custom PCBs offering economical small-batch production. Links to the files deposited with OSHPARK are provided in bill of materials. For three of the boards (the LED connector board (PC3) and the two LED module boards (PC4 and PC5)) special jigs have been designed to aid placement of the components. Most of the components mounted on the PCBs are connected via leads and through-hole connections on the boards, making assembly relatively straightforward. The only surface mount components used are female headers (on PC3); capacitors (on the sensor board, PC2); and resistors (on the LED module boards, PC4 and PC5). Assembly of PC3 is aided by a jig, and the surface mount capacitors and resistors can be mounted by hand, using tweezers and a soldering iron. Although all of the boards are relatively simple, some prior experience in soldering components to PCBs is strongly recommended.

**CAUTION: Although the spectrophotometer is designed to operate at low voltages and currents, resulting in very low electrical hazards, incorrect wiring could lead to short circuits, causing overheating and fire hazards, as well as potential damage to connected devices. Special attention should be given to the polarities of the two tantalum capacitors (C1 and C2) when soldering them to PC1. All of the circuit boards and cable assemblies should be carefully inspected for correct wiring before any of the assemblies are connected to a power supply.**

### 5.2 Circuit boards

#### 5.2.1 PC1: Arduino shield

**Fig. 6:**
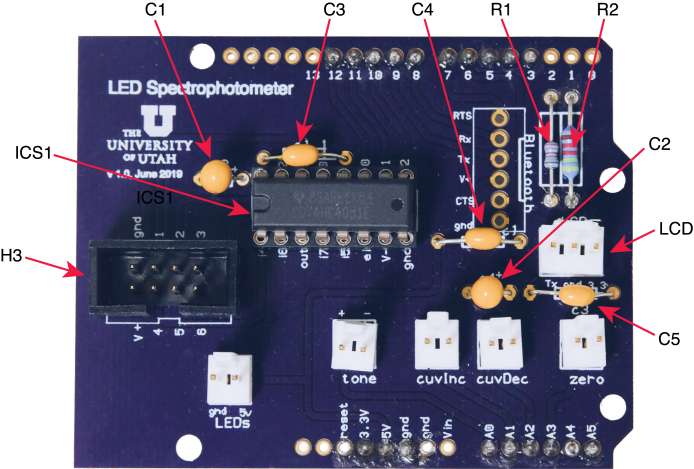
Top side of the Arduino shield, PC1, indicating placement of components.

In the following, positions refer to the board as viewed from the top, with the lettering upright, as in Fig. 6.

1. Solder resistors R1 (22 KΩ) and R2 (44 KΩ) to PC1.
2. Solder the two 1μF tantalum capacitors (C1 and C2) to PC1. These capacitors are polarized and the positive leads must be soldered to the connections labeled “+” on the circuit board.
3. Solder the three 0.1 μF disc capacitors (C3, C4 and C5) to PC1.
4. Solder the 2 × 8 IC socket (ICS1) to PCI. The marked end of the socket should be oriented towards the left side of the board. Although IC1 can be directly soldered to the board, the use of a socket is recommended to facilitate replacement of a damaged or defective IC.
5. Solder the 2 × 4 shrouded male header connector (H3) to PC1, in the position on the left-hand side of the board labeled “Sensors”. Be sure that the opening for the key of the female connector is oriented towards the front of the board, as oriented in the figure.
6. Solder the 3-position locking Molex header to PC1, in the position labeled “LCD”. The locking tab of the connector must be oriented towards the back of the board.
7. Solder the five 2-position locking Molex headers to PC1, in the positions labeled ”LEDs”, “tone”, “cuveInc”, “cuvDec” and “zero”. For each connector, the locking tab should be oriented towards the back of the board.
8. Solder header pins to the positions corresponding to the following groups of Arduino pins:
  • D3–D7 (upper row)
  • D8–D12 (upper row)
  • 3.3 V, 5 V, gnd (lower row)
  • A0–A5 (lower row)
  Although a full complement of Arduino header pins can be soldered to PC1, using only the positions specified above is recommended, in order to make attachment to and removal from the Arduino board easier. The pins are cut from the 36-pin break-away connector (H5) specified in the bill of materials. The headers are mounted so that the plastic portion and the longer side of the pins is on the bottom side of the board, and the pins are soldered to the top. To facilitate correct alignment, it is helpful to use an Arduino board (or a shield with female headers) as a jig to hold the pins while soldering them to PC1. First insert the long side of the pins into the female headers of the Arduino board, being careful to use the correct positions. Then place PC1 over the Arduino and insert the short pins into the holes of PC1. Before soldering, ensure that the plastic portion of the headers are in contact with the bottom of PC1.
9. Insert the 74HC4051 multiplexer IC (IC1) into the IC socket, with the marker oriented towards the left side of the board. This may require bending the IC pins slightly inward. Be sure that the pins are fully inserted in the socket.

#### 5.2.2 PC2: Sensor board

**Fig. 7:**
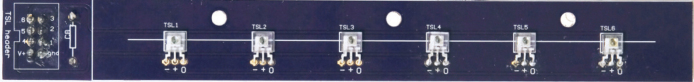
Top side of the sensor board (PC2), with the six TSL235R sensors mounted. The 2 × 4 shrouded male header connector (H3), capacitor C6 (0.1μF) and capacitors C7–12 (0.1μF surface mount) are mounted on the bottom of the board.

In the following, the side of the board on which the TSL235R sensors are mounted is referred to as the top (Fig. 7).

1. Mount and solder the six TSL235R sensors (LS1–LS6) to the *top* of PC2. When mounting these components, the leads should be carefully bent to place the lenses of the sensors as close as possible to the intersecting lines on the board.
2. Mount and solder the 2 × 4 shrouded male header connector (H3) to the *bottom* of PC2. Be sure that the opening for the key of the female connector is oriented towards the edge of the board *away from the sensors*.
3. Solder the 0.1μF disc capacitor (C6) to the *bottom* of PC2.
4. Solder the six 0.1μF surface-mount capacitors (C7–C12) to the *bottom* of PC2. To solder these components by hand, first melt a small bead of solder on one of the two pads. Then, use tweezers to place the capacitor in position, and solder the component in place by reheating the solder. Then solder the other side of the capacitor to its pad.

#### 5.2.3 PC3: LED connector board

This board is used to hold surface-mount female header connectors, which connect to the LED modules. The positions of the connectors must match precisely those of the pins on the LED modules and the openings milled in the cover for the board. To ensure proper alignment, a special jig (milled from ACP) is used.

**Fig. 8:**
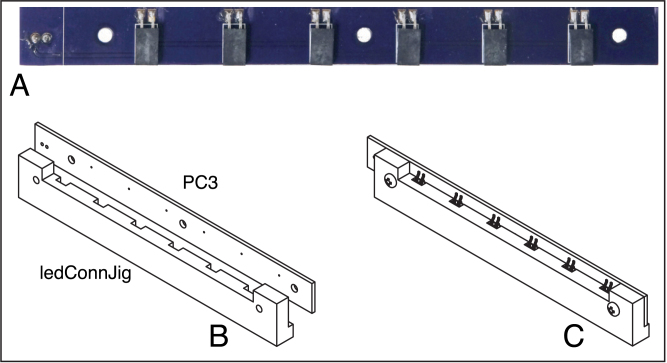
LED connector board (PC3) and jig for soldering surface mount connectors. A. Top side of the board with connectors mounted. B. Board and jig oriented for assembly. C. The assembled jig, board and connectors. A 2-position locking Molex header is soldered to the back of the board. The part labels correspond to the designators in the Bill of Materials and the design-file names listed in Sections 3.2.1–3.2.4.

In the following, the side of the board with the solder pads for the female connectors is referred to as the top, as shown in Fig. 8A.

1. With the top of the board facing upward, attach the assembly jig to the board using 4–40 machine screws and nuts, oriented as illustrated in Fig. 8B and C. Then, slip the six female connectors into the openings of the jig, with the connectors oriented to place the leads into contact with the solder pads on the board. Solder the connectors to the board and then remove the board from the jig.
2. Solder the 2-position locking Molex header to the *bottom* of PC3. The locking tab of the connector should be placed closest to the back edge of the board, as oriented in Fig. 8A (the edge nearest the solder pads for the connectors).

#### 5.2.4 PC4: LED module board for one LED

**Fig. 9:**
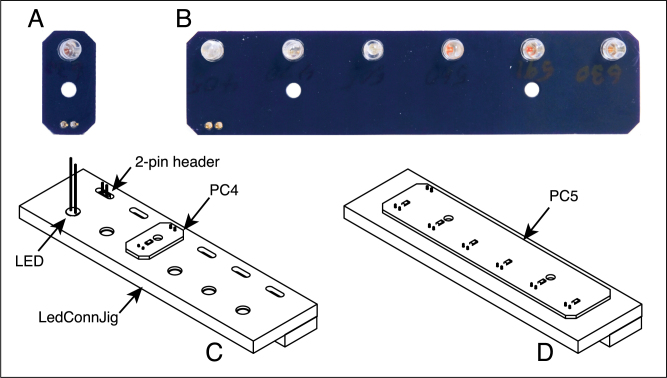
LED module boards and the jig for their assembly. A and B. Boards PC4 and PC5, for one and six LEDs, respectively, with the LEDs and connector pins mounted. C. The assembly jig, as used for the single LED boards. On the left side of the jig, an LED and 2-pin connector are placed in the recesses of the jig. The circuit board is then placed over the pins and LED leads, which are soldered to the back of the board, along with a surface-mount voltage-dropping resistor, as shown in the third position from the left. D. The assembly jig shown with a board for six LEDs. The part labels correspond to the designators in the Bill of Materials and the design-file names listed in Sections 3.2.1–3.2.4.

The jig illustrated in Fig. 9 is used to facilitate assembly of the circuit boards holding the LEDs. This jig is made up of two pieces of 0.25-in thick phenolic resin laminate glued together with a thin layer of epoxy, as illustrated in the figure. In the top piece, cavities are machined to accept the LEDs and two-position male header connectors. The lower layer serves to position the ends of the header pins. After the components are soldered to the board, the plastic part of the header is removed from the pins.

To assemble PC4, with a single LED:

1. Place a 2-pin header in one of the elongated cavities near the back of the jig, as oriented in Fig. 9C. The plastic part of the header will protrude above the jig. Place the circuit board over the pins and use the board to press the plastic part down until it is flush with the jig. Remove the board for now.
2. Place the LED in the round opening below the header. Be sure that the longer (positive) lead is placed towards the left (as oriented in the figure) and the flat surface on the LED lip is towards the right.
3. Place the circuit board over the header pins and the LED leads. The side of the board with the solder pads for the surface mount resistor should be facing upward, and these pads should be closer to the LED than the pins.
4. Solder the pins and LED leads to the circuit board. Trim the leads and pins.
5. Solder a surface-mount resistor (R3; 330 or 660 Ω) to the circuit board. To solder this component by hand, first place a small bead of solder on one of the two pads. Then, use tweezers to place the resistor in position, and solder the component in place by reheating the solder. Then solder the other side of the capacitor to its pad. For most LEDs that have been tested in the spectrophotometer, a 330 Ω resistor has been found appropriate, but the NIVAR 402 nm LED has a significantly higher output, and a 660 Ω resistor should be used.
6. Remove the circuit board from the jig.
7. Carefully remove the plastic part of the header from the pins. A pair of diagonal wire cutters can be used to move the plastic part along the pins. It may be necessary to straighten the pins after removing the plastic part.

#### 5.2.5 PC5: LED module board for six LEDs

The circuit board for holding six LED is assembled using the same jig as for a single LED (Fig. 9D). Only a single 2-pin header is used, but six individual resistors (R3; 330 Ω or 660 Ω) are soldered to the board. The components are assembled and soldered as described above for PC4.

### 5.3 Cables

**Fig. 10:**
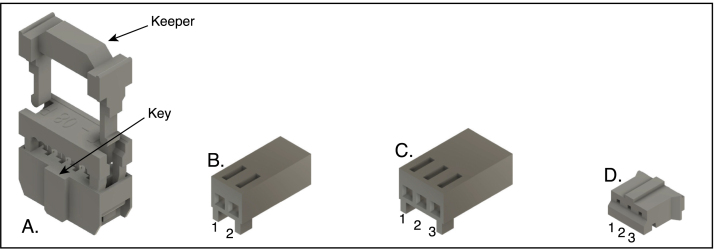
Connector types used for cables in the LED spectrophotometer. A. Insulation-displacement connector (IDC) used for the ribbon cable connecting the Arduino shield (PC1) to the sensor board (PC2). B and C. Molex KK 254 female wire housings for two and three conductors, respectively. D. JST PH female connector. In B and C, the drawings are oriented with the openings for the mating pins facing towards the lower left, and the wires with crimp connectors are inserted from the rear. The pin positions are numbered for reference in the text. The connectors are shown at a common scale.

The three types of cable connectors used in the design are illustrated in Fig. 10:

• 2 × 4-pin ribbon-cable connectors (Fig. 10A). This connector is of the insulation-displacement (IDC) kind, in which forked contacts pierce the insulation of each conductor to form a connection as the two parts of the connector are squeezed together to clamp the cable. The connectors are supplied with separate keepers that help hold the cable in place after being folded over the top of the connector (as oriented in the figure). Video instructions for attaching this type of connector can be found on the internet, such as Ref. [26]. Although special crimpers are available for this purpose, the small connectors used in this application can be reliably squeezed using a small vise (*e.g.,* PanaVise).
• Molex KK 254 (or similar) female connectors (Fig. 10 B and C), which attach to matching headers on the Arduino shield and the LED connector board. Connectors of this type are assembled by first crimping individual connectors to each connector and then inserting the connectors into a wire housing. Video instructions for crimping and assembling this type of connector can be found on the internet, such as ref [27]. If very many connectors of this type are to be used, the Molex crimping tool (model 63 811–8200) is recommended. There are, however, much less expensive options, such as the IWISS SN-2549, that can work well if used carefully [28].
• JST (Japan Solderless Terminal) PH female connector (Fig. 10A), used for the LCD board. This connector is similar in design to the Molex KK type, but is considerably smaller and more difficult to work with. As only one connector of this type is used in the design, it is recommended that it be purchased with pre-attached wires.

#### 5.3.1 8-Conductor ribbon cable, from Arduino shield to sensor board

The correct orientation of the connectors on this cable is critical to the proper functioning of the instrument and is illustrated in Fig. 10. The polarization of the connector is determined by a key, indicated in the figure, that fits into an opening in the header shroud. Many, but not all, connectors of this type are also marked with an arrowhead to indicate the orientation of the connector, or a mark may be found on the mating header connector.

**Fig. 11:**
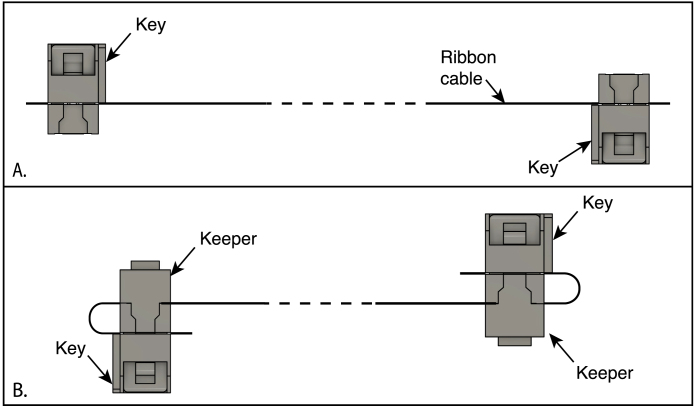
Orientation of connectors on the ribbon cable used to connect the Arduino shield to the sensor board, as viewed from one edge of the cable. A. After attaching the connectors, but before adding the keepers. B. After folding the cable over the tops of the connectors and attaching the keepers. Note the positions of the keys at the two cable ends.

1. Cut a 25 cm length of 8-conductor ribbon cable. The cable listed in the bill of materials is made up of 16 conductors, but can be easily split into narrower pieces. (Not all ribbon cable can be split in this way.)
2. Thread one end of the ribbon cable between the two sections of one of the connectors, allowing about 6 mm to extend beyond the connector. The key of the connector should be pointed away from the cable end. After checking that the conductors of the cable are properly aligned with the indents in the connector, carefully squeeze the two parts of the connector together.
3. Orient the cable and the second connector as shown in Fig. 11A, and thread the cable through, allowing about 6 mm to extend beyond the connector. The key should point away from the cable end, and the bottoms of the two connectors (with the openings for the mating pins) should lie on opposite sides of the cable. If the connectors are marked with arrowheads, the marks on both connectors should to point to the same edge of the cable. Squeeze the two parts of the connector together.
4. For each connector, fold the cable over the top of the connector (away from the pin openings) and attach the keeper, as shown in Fig. 11B.

#### 5.3.2 2-Conductor LED power-supply cable, from Arduino shield to LED connector board

This cable is constructed from two-conductor, 24 AGW wire, with female Molex KK 254 connectors at each end. The wire housings are polarized, as shown in Fig. 10B, and must be assembled in the same orientation at the two ends of the cable.

1. Cut a 15 cm length of 2-conductor 24 AGW wire and strip 3 mm of insulation from both conductors at each end.
2. Crimp one of the metal crimp connectors onto each of the four wire ends.
3. Insert the crimp connectors at one end of the cable into the two positions of one of the wire housings, from the back.
4. Insert the crimp connectors at the other end of the cable into the second housing, taking care to ensure that the polarities of the two ends are the same: The conductor in position 1 of the first connector (as shown in Fig. 10B) should be inserted into position 1 of the other connector.

#### 5.3.3 3-Conductor cable, from arduino shield to LCD board

The LCD board is connected to the Arduino board via a three-pin JST PH connector. Item JST in the BOM includes both the male and female connectors, with wires already attached to the female connector. The other end of the cable uses a 3-position female Molex KK 254 connector (Fig. 10C). To facilitate the final assembly it is suggested that the wires that come with the JST connector be extended to a total length of 20 cm, if necessary.

**Fig. 12:**
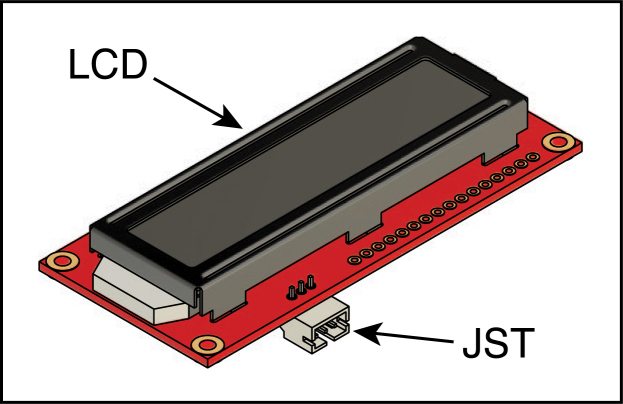
The LCD board, illustrating the position of the three-pin JST connector.

1. Strip 6 mm of insulation from each of the three wires already connected to the JST female connector.
2. Prepare three pieces of 24 AWG wire, of a length sufficient to extend the wires already attached to the JST connector to a total of 20 cn, and strip 6 mm of insulation from one end of each. Splice and solder one of the pieces of wire to each of the wires attached to the JST connector. Cover the splices with heat-shrink tubing.
3. Strip 3 mm of the free end of each wire and crimp one of the Molex crimp connectors to each of the wire ends.
4. Insert the three connectors into the Molex wire housing from the back. The wires should be inserted so that they occupy the same numbered positions at each end of the cable, using the numbering scheme shown in Fig. 10C and D.
5. Solder the three-pin male JST connector to the LCD board, as illustrated in Fig. 12. The board contains two sets of holes for soldering connectors; a set of three holes separated by 2 mm, and a set of 16 separated by 2.54 mm (0.1 in). The set of three holes (labeled ‘+’,‘–’ and ‘RX’) are used for the JST connector. The connector is attached to the bottom of the board, with the connections facing away from the board, and soldered from the top.

#### 5.3.4 2-Conductor cables (3) for push-button switches

These cables are constructed from two-conductor, 24 AGW wire, with female Molex KK 254 connectors at one end. The other end is soldered directly to a push-button switch. The soldered connections are covered with heat-shrink tubing to provide additional physical strength, as well as to prevent possible short circuits.

For each of the three cables:

1. Cut a 15 cm length of 2-conductor 24 AGW wire and strip 3 mm of insulation from both conductors at one end of the wire.
2. Attach a two-position female Molex connector on the stripped end of the wire, as described in Section 5.3.2.
3. Strip 6 mm of insulation from each wire at the free end of the wire.
4. Slip a 10 mm length of 2 mm diameter heat-shrink tubing over each of the free conductors, moving the tubing at least 15 mm from the end.
5. Solder the wire ends to the two terminals of the push-button switch.
6. Slide the heat-shrink tubing over the solder joints and terminals. Using a heat gun or other device, apply hot air (approximately 90 °C for polyolefin tubing) to the heat-shrink tubing.

#### 5.3.5 2-Conductor cable for buzzer

This cable connects a small magnetic buzzer to the Arduino shield and is constructed as described above for the cables on the push-button switches. Unlike like the switches, however, the speaker is polarized. The terminal labeled ‘+’ on the speaker should be connected to position 1 of the connector, as labeled in Fig. 10B. After soldering the wires to the buzzer, the connections should be covered with heat-shrink tubing.

#### 5.3.6 USB cable with power switch

This cable connects a panel-mounted female USB connector to the Arduino board and incorporates a power switch for the instrument. The panel-mounted connector is used to provide power and a USB data connection. This part is based on a commercially available cable, modified to accommodate the power switch. The Arduino-compatible board listed in the bill of materials (SparkFun RedBoard) includes a USB Mini-B female connector, and the listed cable has a matching USB mini-B male connector at one end and a panel-mounted female USB B connector at the other. Cables with different USB connector types are available for use with other boards, or if a different external connection is desired. If a different panel-mounted connector is used, it may be necessary to modify the design of the rear panel.

The cable is modified and connected to the power switch as follows:

1. Starting 2.5 cm from the panel-mount connector, carefully remove 4 cm of the outer insulation from the cable. Then, separate the metal-foil or braided shielding to expose the four wires. Draw the positive power wire (with red insulation) away from the other wires and the shield. Cut this wire and remove 6 mm of insulation from the ends.
2. Cut a 10 cm length of 2-conductor 24 AGW wire and strip 6 mm of insulation from both conductors at each end of the wire.
3. Slip a 10 mm length of 2 mm diameter heat-shrink tubing over one of the conductors at one end of the 2-conductor wire. Solder this end to one of the cut ends in the USB power wire.
4. Repeat the step above for the other conductor in the wire and the other cut end of the USB power wire.
5. Slip the pieces of heat-shrink tubing over the solder joints and apply heat.
6. Fold over the 2-conductor wire so that it is parallel with the USB cable and points towards the panel-mount connector. Wrap the junction area with electrical tape.
7. Slip a 10 mm length of 2 mm diameter heat-shrink tubing over one of the conductors at the free end of the 2-conductor wire. Solder this end to one of the toggle switch terminals (SW1).
8. Repeat the step above for the other conductor in the wire and the other switch terminal.
9. Slide the heat-shrink tubing over the solder joints and switch terminals. Apply heat to the tubing.

### 5.4 Loading software and testing the electronic components

Before proceeding with the mechanical assembly of the spectrophotometer, the electronic components should be tested, which requires temporarily connecting them and loading software to the Arduino and LCD panel.

#### 5.4.1 Temporary wiring

All of the connections are made using polarized connectors. In each step, be sure that the connectors are properly oriented before attempting to fit them together. Refer to Fig. 6 for the locations of the various connectors on the Arduino shield board (PC1).

**Fig. 13:**
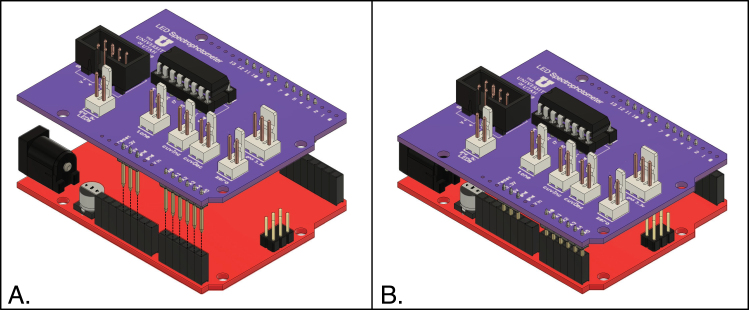
Assembly of the Arduino shield onto the Arduino board.

1. Mount the Arduino shield (PC1) to the Arduino board, as illustrated in Fig. 13. The two boards must be oriented as shown in the figure, and the pins on the shield and the female headers on the Arduino board must be aligned as shown.
2. Temporarily connect the Arduino board to a power supply (either an AC adapter or a computer) using an appropriate USB cable. Immediately after the power is connected, an orange LED on the Arduino board should flash briefly, and a green LED should remain on. If the LEDs do not light correctly, there may be a short circuit somewhere on the shield board. Quickly disconnect the power, remove the shield from the Arduino board and carefully inspect all of the solder connections on the shield. Look for spots where excess solder may have caused a short circuit, and for missing or poor solder connections. Do not continue testing before resolving this issue.
3. Disconnect the power from the Arduino board.
4. Using the 8-conductor ribbon cable, connect the shrouded headers on the shield and the sensor board (PC2). The cable is symmetrical, and either end can be connected to either board.
5. Connect the LCD panel to the shield using the 3-conductor cable prepared in Section 5.3.3.
6. Connect the buzzer to the Molex header on the shield labeled “tone”.
7. Connect the three push-button switches to the three Molex headers on the shield labeled “cuvInc”. “cuvDec” and “zero”.
8. Using the 2-conductor cable with Molex connectors at each end, connect the LED connector board (PC3) to the Molex header on the shield labeled “LEDs”. Then insert the pins of one of the LED circuit boards (PC4 or PC5) into one of the female headers on PC3. The LED board should point in the same direction as the Molex header on PC3, and away from the side of PC3 with the headers.
9. Plug the male Mini-B USB connector of the modified USB cable into the USB connector of the Arduino board. Connect the female USB B connector on the cable to a computer, using a separate USB A to USB B cable.
10. Set the toggle switch connected to the USB cable to the on position. After the power is turned on, a green LED on the Arduino board should be lit, the LCD panel should be illuminated and the LEDs on PC4 or PC5 should be lit. If the LEDs do not light up, immediately disconnect the circuit from its power supply. One potential problem is a short circuit, which could damage one or more components if left connected to power.
11. If any of the LEDs do not appear to be working, there are likely one or more wiring errors that should be corrected before proceeding. The following trouble-shooting steps are suggested:
  • Check that the pins of the Arduino shield are in proper register with the corresponding female headers on the Arduino board and are properly inserted.
  • Carefully check all of the soldered connections on all of the circuit boards.
  • Check that all of the polarized connectors on the boards and cables have been properly installed.
  • Check the connections in all of the cables, using an ohmmeter.

#### 5.4.2 Loading the software

The software for the spectrophotometer is uploaded to the Arduino board via a USB connection to a desktop or laptop computer. Arduino programs are referred to as “sketches”, and two are used with the LED spectrophotometer: LEDspec.ino and serLCDsetup.ino. LEDspec.ino is the sketch used for normal operation of the instrument, whereas serLCDsetup.ino is loaded temporarily to set parameters for the LCD panel. In addition to the sketches, several Arduino libraries are required. The steps required for installing the software are outlined below, with online references provided.

1. Download and install the desktop Arduino IDE (integrated development environment) application. See https://www.arduino.cc/en/Guide/HomePage.^2^
2. Download the zip archive containing the sketches for the spectrophotometer, ledSpec-arduino.zip.
3. Move the zip file to the Arduino folder in the user’s Documents folder. Then, then expand the archive folder.
4. Connect the Arduino board to a USB port on the computer and then open the desktop Arduino IDE application.
5. Check to see if the Arduino board is recognized by the computer:
  • Select **Arduino Uno** in the **Board** → **Arduino AVR** submenu of the **Tools** menu. (Select this option even if using the SparkFun RedBoard or other Uno-compatible boards.) Then, check for the connection to the board by examining the **Port** submenu of the **Tools** menu. On MacOS, the port should be identified by a string of the form “dev/usbserial...”, or similar. On Windows, the port will be identified as “COMMX”, where “X” is an integer. If the port does not appear in the submenu, it may be necessary to load driver software for the board, as described below.
6. If necessary, install driver software for the Arduino board. Before taking this step, however, be sure that a driver is not already present. The drivers for the Arduino Uno board should already be present after installing the Arduino desktop IDE. The driver for the SparkFun RedBoard should already be installed with recent versions of MacOS, and installing a second version will likely cause conflicts. For Windows, however, the driver for the RedBoard and some other boards will have to be installed separately. For information on the drivers for the RedBoard, see: https://learn.sparkfun.com/tutorials/redboard-hookup-guide (See ‘Install FTDI Drivers’.)
  The drivers are available at
  https://www.ftdichip.com/Drivers/VCP.htm.
  For information on setting up the drivers for the Arduino Uno board, see:
  https://www.arduino.cc/en/Guide/ArduinoUno.
7. Download and install the following Arduino libraries:
  • Bounce2 by Thomas Ouellet Fredericks For debouncing and handling button signals https://github.com/thomasfredericks/Bounce2
  • FreqCount by Paul Stoffregen For frequency counting the signal from the TSL235R photodetectors https://www.pjrc.com/teensy/td_libs_FreqCount.html
  • SerialCommand by Seven Cogswell and Stefan Rado For interpreting serial commands from a USB-connected computer
    https://github.com/kroimon/Arduino-SerialCommand
  • TimerFreeTone by Tim Eckel For generating the tone used to indicate cuvette changes https://bitbucket.org/teckel12/arduino-timer-free-tone/wiki/Home
  Libraries can be installed on the local computer either by using the Library Manager (found in Arduino IDE version 1.6.2 and later) or by downloading them from the sites listed above. For detailed instructions, see https://www.arduino.cc/en/Guide/Libraries.
8. Upload the sketch, serLCDsetup.ino, to the Arduino board. From the Arduino IDE program, open the file by selecting it from the **Sketchbook** submenu in the **File** menu. Upload the program by selecting the **Upload** command in the **Sketch** menu, or by clicking the upload button (a circle with an arrow pointing rightward) in the sketch window. This sketch uses the Arduino as an intermediary to send commands to the microprocessor on the LCD board, and these commands set the contrast and background color for the LCD and define a splash screen. The sketch will run automatically after being loaded to the Arduino, and the splash screen should appear in the display:
  LED Spec, Univ of Utah, 2019
  The color parameters and splash screen can be modified by editing the sketch.
9. Upload the sketch, LEDspec.ino, following the same procedure as above. After the sketch is uploaded, it should begin running. The splash screen should appear briefly on the LCD, followed by messages indicating that the values for the individual cuvette positions are being zeroed. After this process is complete, the LCD should display:
  Abs: 0.000 Cuvette 1
  However, because the sensors are exposed to ambient light and are very sensitive to position, the values displayed may shift significantly.

#### 5.4.3 Testing the software and electronics

1. Check the functions of the three buttons and the buzzer:
  • Pressing the cuvInc button should increase the cuvette number indicated in the display, until the value of 6 is reached, when the number will return to 1. With each change in cuvette number, the buzzer will beep.
  • If the cuvette number displayed is greater than 1, pressing the cuvDec button will decrease the number until the value of 1 is reached. The number will then change to 6.
  • Pressing the Zero button, will set the absorbance value displayed for the indicated cuvette position to 0.
2. Check that each of the six sensors is correctly responding to light intensity. This can be done by stepping through the cuvette positions, using the cuvInc or cuvDec button, and temporarily blocking from light the corresponding sensor on PC2. The displayed absorbance value should increase when the sensor is blocked and decrease when it is exposed.
3. If any of the functions described above to not appear to work properly, carefully check the circuitry.
4. Once the functions of the circuit has been confirmed, disconnect the various cables, but leave the Arduino and shield boards mated.

### 5.5 Core assembly

The overall design of the core assembly is illustrated in Fig. 3. The following instructions assume that the plastic parts have been machined using the three-dimensional CAD files provided (but the holes not yet threaded) and the circuit boards (PC2 and PC3) have been assembled, as described in Sections 5.2.2, 5.2.3.

#### 5.5.1 Threaded holes

The components of the core assembly are held together with hex socket-head screws, threaded into the two halves of the cuvette block and the LED block. For the threaded holes most likely to suffer wear from repeated assembly and disassembly, helical threaded inserts (Heli-Coil® or equivalent) are used. Installation of these inserts requires a special tap and insertion tool for each size (4–40 and 6–32). Because the ACP plastic is relatively soft, care must be taken to make sure that the taps and inserts are inserted correctly, and excessive force should be avoided.

**Fig. 14:**
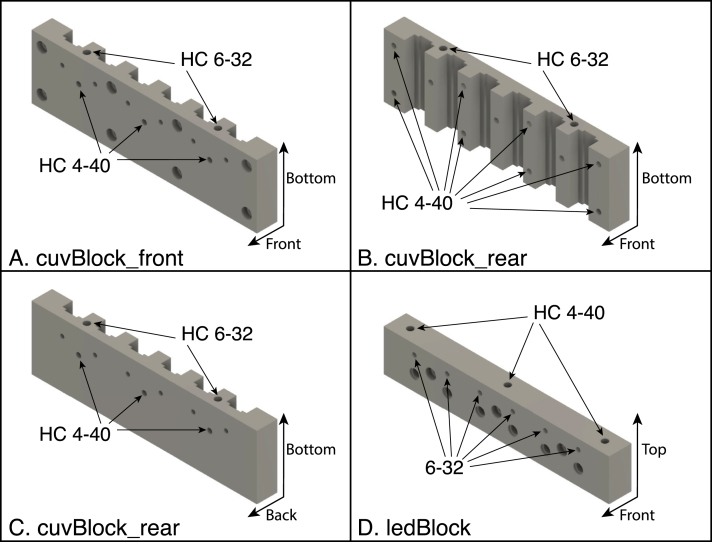
Positions of threaded holes in the front and rear cuvette-block halves (A, B and C) and the LED block (D). In each panel, the coordinate arrows at the lower right indicate the orientation of the components in the assembled instrument. The holes labeled “HC 4-40” and “HC 6-32” are threaded with special taps for the helical inserts for the indicated sizes. The holes labeled “6-32” on the LED block are tapped directly with a tap of that size. The part labels correspond to the designators in the Bill of Materials and the design-file names listed in Sections 3.2.1–3.2.4.

The positions of the tapped holes are illustrated in Fig. 14. Note that some of the holes pass completely through the components, and the inserts should be installed from the sides indicated in the drawings. The holes are tapped and inserts installed as follows:

1. Tap threads for 4–40 helical inserts in the following components and install the inserts:
  • Cuvette block, front half: The three holes labeled “HC 4-40” in Fig. 14A.
  • Cuvette block, rear half: Eight holes in the front of the component, as oriented in Fig. 14B.
  • Cuvette block, rear half: Three holes in the back of the component, (facing forward in Fig. 14C).
  • LED block: Three holes at the top (Fig. 14D)
2. Tap threads for 6–32 helical inserts in the following components and install the inserts:
  • Cuvette block, front half: Two holes at the bottom of the component as oriented in the assembled instrument (oriented at the top in Fig. 14A).
  • Cuvette block, rear half: Two holes at the bottom of the component as oriented in the assembled instrument (oriented at the top in Fig. 14B).
3. Tap 6–32 threads (not for inserts) in the six holes labeled “6-32” at the front of the LED block (Fig. 14D).

#### 5.5.2 Assembly

**Fig. 15:**
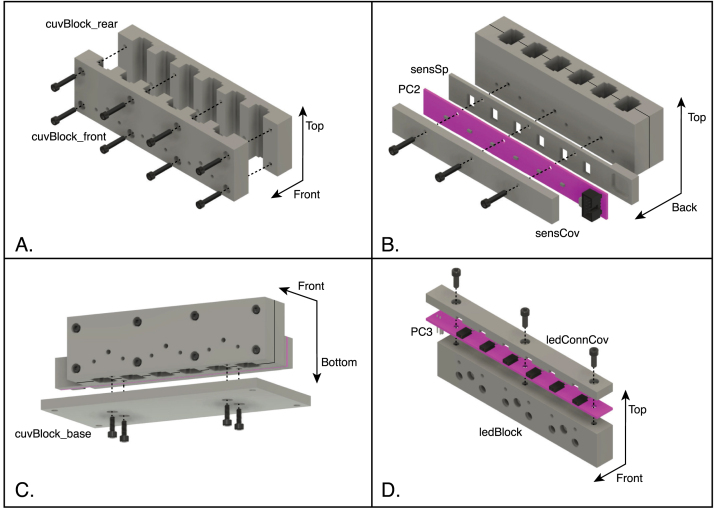
Assembly of the components making up the spectrophotometer core assembly. A. Assembly of the two halves of the cuvette block. B. Assembly of the sensor spacer, sensor PCB (PC2) and sensor cover onto the back of the cuvette block. C. Attachment of the cuvette-block base to the bottom of the assembled cuvette block. D. Assembly of the LED connector board (PC3) and connector cover to the LED block. The coordinate arrows indicate the orientation of the components in the assembled instrument. The part labels correspond to the designators in the Bill of Materials and the design-file names listed in Sections 3.2.1–3.2.4.

The steps in the assembly of the spectrophotometer core components are listed below and illustrated in Fig. 15. Note that many of the components are highly, but not completely, symmetrical, and care must be taken to ensure that they are assembled in the correct orientation. All of the screws referred to in these steps are of the hex socket-head type.

1. Using eight 4-40×58 in screws, assemble the two halves of the cuvette block to one another, as illustrated in Fig. 15A. The tops and bottoms of these components are distinguished by the mounting holes on the bottom surfaces, and the two halves must be assembled so that these holes are on the some side. (See Fig. 14A and B.)
2. Arrange the sensor spacer, the sensor PCB (PC2) and sensor cover as oriented in Fig. 15B. Use three 4-40×38 in screws to attach these components to the rear side of the cuvette block.
3. The cuvette-block base has two sets of four counterbored holes, one set at the outer corners of the base and the other closer to the center and placed to align with the mounting holes on the bottom of the cuvette block. With the counter bores of the more central set of holes facing away from the cuvette block, as illustrated in Fig. 15C, use four 6-32×38 in screws to attach the base to the cuvette block.
4. Arrange the LED block, the LED connector board (PC3) and the connector cover as shown in Fig. 15D. The openings in the connector cover should mate with the connectors on the circuit board, and the openings of the connectors should face the front of the LED block, as oriented in the figure and in the assembled instrument. (The two sides of the LED block are distinguished by sizes of the holes drilled in them, with the larger holes on the front.) Attach the connector board and cover to the LED block using three 4-40×38 in screws. The EPS file, cuvPosLabels.eps, can printed onto self-adhesive paper stock to produce a label to be placed on the LED cover, identifying the six cuvette positions in the completed spectrophotometer. Do not attach the LED block assembly to the cuvette block at this time, as doing so will block access to the holes in the base used to attach the assembly to the enclosure bottom.

### 5.6 LED modules

The LED modules (Fig. 4) consist of the printed circuit boards (PC4 or PC5), the matching covers (machined from phenolic resin laminate) and modified nylon thumbscrews.

1. After machining the LED module covers, tap the holes for 6–32 threads. The cover for a single LED has one hole, whereas the cover for the 6-LED circuit board has two holes
2. After assembling the circuit boards (Sections 5.2.4, 5.2.5), use cyanoacrylate glue to attach the printed circuit board to the corresponding cover. The covers for the circuit boards are not symmetrical, and care must be taken to align them so that the cutouts accommodate the components on the corresponding circuit boards, as shown in Fig. 4. Apply the glue sparingly to only a few spots on the edge of the cover that contact the circuit board, so that the cover and circuit board can be separated if needed at a later time.
3. Using a lathe, modify the thumbscrews by turning down the threads from the region (6 mm long) closest to the head, as shown in Fig. 4. With this modification, the screws thread into the module covers and are captured, but rotate freely.
4. The EPS file, LEDlabels.eps, can be used to print labels on self-adhesive paper, to identify the modules by the LED wavelengths. The file contains templates for single-LED modules and six-LED modules.

### 5.7 Enclosure

The instrument enclosure is composed primarily of 3/8 in thick black acrylic sheet, as illustrated in Fig. 5 and detailed below.

#### 5.7.1 Threaded holes

**Fig. 16:**
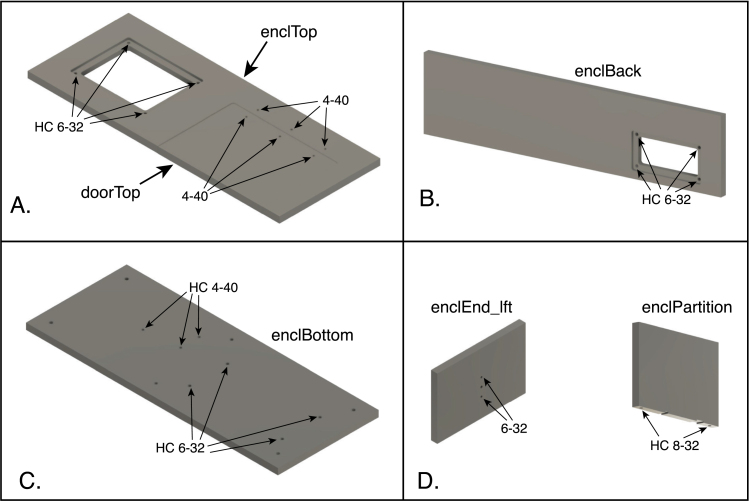
Positions of threaded holes in components of the enclosure. (A) The enclosure top and the top part of the door, shown from the top. (B) The enclosure back panel, shown from the back. (C) The enclosure bottom, shown from the top. (D) The left panel, shown from the inside, and the partition separating the cuvette chamber from the electronics. The holes labeled “HC 4-40”, “HC 6-32” and “HC-8-32” are threaded with special taps for the helical inserts for the indicated sizes. The holes labeled “4-40” on the enclosure top and “6-32” on the left panel are threaded directly with a tap of the indicated size. The part labels correspond to the designators in the Bill of Materials and the design-file names listed in Sections 3.2.1–3.2.4.

Some of the acrylic parts making up the enclosure are threaded to allow the attachment of other components. The positions of the threaded holes are indicated in Fig. 16, and detailed below. Refer also to Fig. 3 for the orientations of the components in the assembled enclosure. Some of the holes pass entirely through the panels, and inserts in these holes should be installed from the side indicated in the figures.

1. Tap 4–40 threads (not for inserts) in the following components:
  • Enclosure top: Three holes at the rear right-hand side of the panel (Fig. 16A).
  • Door top: Three holes at the rear of the panel (Fig. 16A).
2. Tap 6–32 threads (not for inserts) in the upper and lower holes on the inner side of the left panel (Fig. 16D).
3. Tap threads for 4–40 helical inserts in the three holes on the left side of the enclosure bottom (Fig. 16C) and install the inserts.
4. Tap threads for 6–32 helical inserts in the following components and install the inserts:
  • Enclosure top: four holes in the recess for the aluminum display panel, at the right side of the top panel(Fig. 16A).
  • Enclosure back: four holes in the recess for the rear aluminum panel (Fig. 16B).
  • Enclosure bottom: four holes on the right side of the panel (Fig. 16B).
5. Tap holes for 8–32 helical inserts in the following components and install the inserts:
  • Enclosure partition: two holes on the bottom edge of the panel (Fig. 16D).
  • four blocks to be placed in the lower corners of the enclosure. (See Fig. 5.)

#### 5.7.2 Assembly of the enclosure

The acrylic panels making up the enclosure are glued together, piece by piece, using a solvent cement, such as IPS Weld-On® 4. Cements of this type act by softening the surfaces that are to be attached, so that they are fused when pressed together and the solvent evaporates. The bond begins to form within a few minutes, and increases in strength over the next several hours. Care must be taken to carefully position the pieces and hold them as the bond forms. Although the cement can be applied to the surfaces before placing them in contact, it is recommended that the pieces first be held together and the cement applied along the edges of the joint, using a syringe with a needle or other fine-tipped applicator. Capillary action then draws the cement between the two surfaces. After the cement has flowed between the pieces, apply moderate pressure for a few minutes. To form square joints between two panels, place one piece on a flat surface and hold the other upright using one or more blocks to hold it in position.

**CAUTION: Weld-On® 4 and similar cements contain methylene chloride, a volatile, flammable, and highly toxic compound. Before using the cement, carefully read and follow the manufacture’s instructions and the safety data sheet.**

**Fig. 17:**
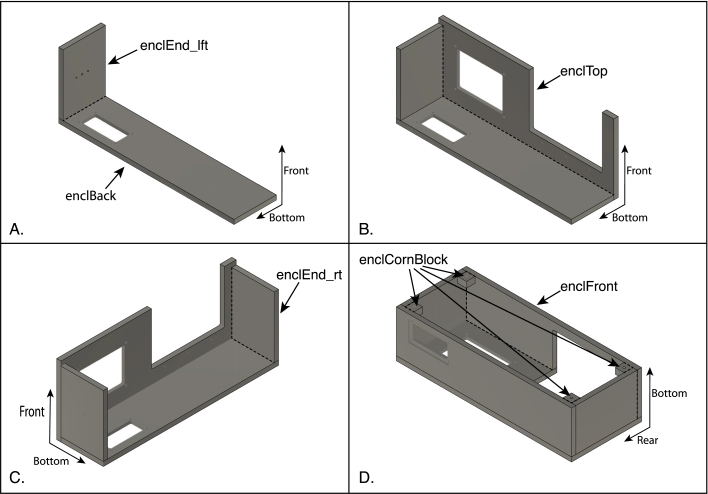
Assembly of the acrylic panels making up the enclosure. The panels are glued together using a solvent cement. In each step, one of the panels is placed flat on a table surface and the second is held upright using blocks. The two pieces are first placed in position without cement, which is then applied to an edge of the joint (highlighted with dashed lines in the drawings) using a syringe and needle or other fine-tipped applicator. (A) The back and left-end panels are cemented together first. (B) The top panel is glued to the back and left panels. (C) The right-end panel is added to the assembly. (D) The front panel is added, followed by four corner blocks that are used to attach the enclosure to its base with screws. In (D) portions of the seams between the front panel and the top and right panels are hidden from view by the right panel. In each drawing, the coordinate labels indicate the orientation of the pieces in the assembled instrument. The part labels correspond to the designators in the Bill of Materials and the design-file names listed in Sections 3.2.1–3.2.4.

**Fig. 18:**
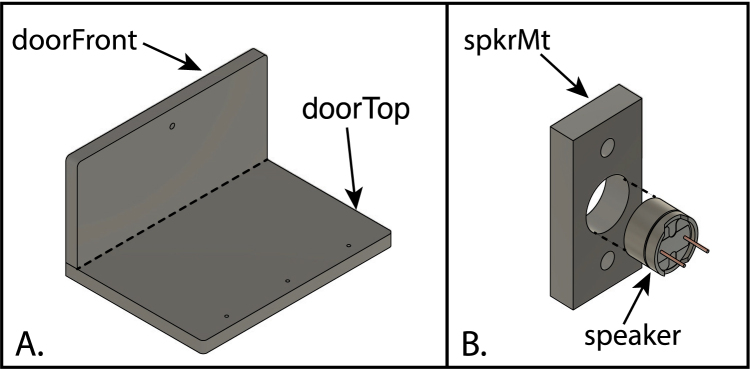
Assembly of the two acrylic panels making up the enclosure door (A) and attachment of the buzzer to its mount (B). In A, the edge of the seam between the two door pieces, where solvent cement is applied, is highlighed by a dashed line. The buzzer is inserted into the opening in the mount and fastened using a drop of cyanoacrylate glue. The part labels correspond to the designators in the Bill of Materials and the design-file names listed in Sections 3.2.1–3.2.4.

A suggested sequence for assembling the piece of the enclosure is illustrated in Fig. 17, Fig. 18, and detailed below. In each of the drawings, dotted lines are used highlight the edges of the joints to which the cement is applied, and coordinate labels indicate the orientation of the pieces in the assembled instrument. After each step, allow the joint to harden (30–60 min) before moving on to the next.

1. Glue the left-end panel to the rear panel (Fig. 17A). Note, first that the left- and right-end panels are distinguished by the three holes in the left panel, which are for the small speaker to be attached later. The central hole passes all the way through the panel, whereas the two outer holes are only exposed on one side and were tapped for 6–32 screws in an earlier step. The panel should be oriented so that the holes will be placed closest to the bottom of the enclosure and the tapped holes face inward, as shown on the drawing. Also note that the rear panel has an opening for an aluminum panel to be installed later, and there is a recess surrounding the opening, which faces outward. The end panel is placed so that its rear edge is cemented to the inner surface of the rear panel.
2. Glue the top panel to the rear and left panels (Fig. 17B). Like the rear panel, the top panel has a cutout for an aluminum panel (on the left in the figure), with a recess that faces outward. The upper edges of the rear and left panels are cemented to the inner surface of the top panel.
3. Glue the right-end panel to the rear and top panels (Fig. 17C). The rear and upper edges of the right panel are cemented to the inner surfaces of the rear and top panels, respectively.
4. Glue the front panel to the assembly (Fig. 17D). The upper edge of the front panel is cemented to the inner surface of the top panel, and the front edges of the left- and right-end panels are glued to the inner surface of the front panel.
5. Glue the four corner blocks into the corners of the enclosure (Fig. 17D). The blocks should be oriented so the threaded inserts face the bottom of the enclosure, and the blocks should be flush with the bottom edges of the front, rear and end panels. When gluing these parts, first apply the cement to the two surfaces of the blocks that will mate with the enclosure panels, and then place them in position. Hold the blocks in place for a few minutes until the joint has begun to harden.
6. Glue the two pieces of the door assembly together (Fig. 18A). The two panels making up the door are distinguished by the three threaded holes for the door hinge in the top panel and the single hole for a knob in the front panel. The top edge of the front panel is glued to the inner surface of the top panel. After the joint is fully hardened, attach the knob to the door with a 6-32×58 in pan-head screw, with the knob facing outward.
7. Glue the magnetic speaker to its mounting piece (Fig. 18B). For this step, solvent cement should NOT be used, as it may damage the speaker. Instead, use a drop of cyanoacrylate glue applied to the buzzer. The front of the buzzer should be flush with the surface of the mount. The two pieces must be assembled quickly, or the cement may set before the buzzer is fully inserted.
8. Using two 6-32×12 in in cap-head screws, attach the buzzer and mount to the inner side of the left-end enclosure panel.

### 5.8 Control and display panel

**Fig. 19:**
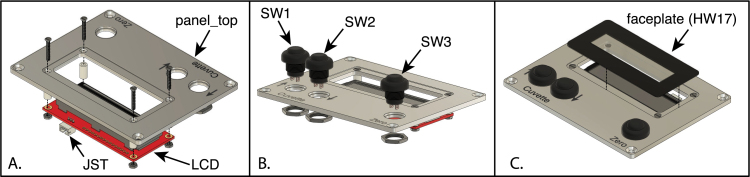
Assembly of the control and display panel. (A) Attachment of the LCD board to the panel, using 2–56 screws and nuts, as provided in the mounting kit from Seetron.com. (B) Mounting the three push-button switches to the panel, using nuts provided with the switches. Note that the wires connecting the switches to the Arduino board are not shown here. (C) Attachment of the faceplate to the aluminum panel. Panel C also shows the labeling applied to the panel. The part labels correspond to the designators in the Bill of Materials and the design-file names listed in Sections 3.2.1–3.2.4.

The LCD display and three push-buttons for control of the spectrophotometer are mounted to an aluminum plate that is, in turn, mounted to the top of the enclosure. The LCD circuit board is attached to the panel using hardware provided in a mounting kit from Seetron.com, which also includes a Lexan faceplate for the LCD. The assembly of the panel components is illustrated in Fig. 19 and detailed below.

1. The push-buttons on the panel should be labeled, as shown in Fig. 19C, and a variety of methods can be used, including engraving, vinyl cutouts, adhesive labels or water-transfer decals. A layout of the labels is provided as file, topLabel.eps. Depending on the method used, it may be preferable to apply the labels before or after installing the LCD board and push-button switches on the panel. If inkjet-printed labels or decals are used, they should be applied, and the panel sprayed with a clear acrylic finish, before assembly.
2. Mount the LCD board to the aluminum panel, using four 2-56×58 in screws, four 0.375 in long plastic spacers and four 2–56 hex nuts, as illustrated in Fig. 19A. Ensure that the board is correctly oriented, with the JST connector pointing away from the switches. Before mounting the LCD, remove the protective film from it surface.
3. Attach the three push-button switches to the panel, using the nuts provided with the switches (Fig. 19B). There may not be room for the lock-washers provided with the switches, and these are not necessary.
4. Attach the faceplate to the recessed surface of the aluminum panel (Fig. 19C). The faceplate is provided with an adhesive backing that is covered with a protective paper, as well as a clear protective film on the top surface. Before removing the backing paper, trim the corners of the faceplate to match the rounded corners of the recess. Then, remove the backing and carefully align the faceplate with the recess and press it in place. It is recommended that the protective film on the outer surface be left in place until assembly of the spectrophotometer is complete.
5. Connect the JST cable (prepared in Section 5.3.3) to the connector on the LCD board.

### 5.9 Rear panel insert

**Fig. 20:**
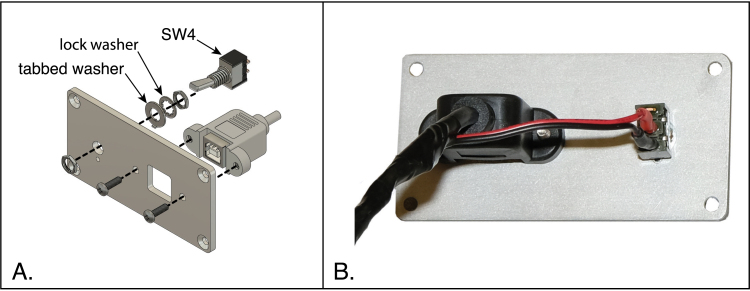
Assembly of the rear panel, with USB port and power switch. (A) Exploded view of the panel components and mounting hardware. (B) Photograph of the assembled components.

A second aluminum panel is used to mount the USB port and power switch to the rear of the instrument. The USB port and switch are wired together, as detailed in Section 5.3.6. The assembly of these components is illustrated in Fig. 20 and detailed below. The hardware shown is provided with the switch and USB cable.

1. Fully screw one of the two hex nuts onto the switch bushing. This nut is followed by the lock washer and the tabbed washer. Note that this second washer has two tabs, one on the inner diameter, which mates with a groove on the switch bushing, and an outer tab. The outer tab should face away from the switch.
2. Orient the switch and panel as shown in Fig. 20A (so that the countersinks on the four corner holes face away from the switch), and insert the switch bushing into its hole. Be sure that the outer tab of the tabbed washer fits into the small hole in the panel. Then fasten the switch to the panel using the second hex nut.
3. Mount the USB connector to the panel, using the two M3 screws provided with the connector. The tapered portion of the opening in the USB connector should face the top of the panel (as oriented in the drawing and in the final, assembled instrument.)

### 5.10 Final assembly

**Fig. 21:**
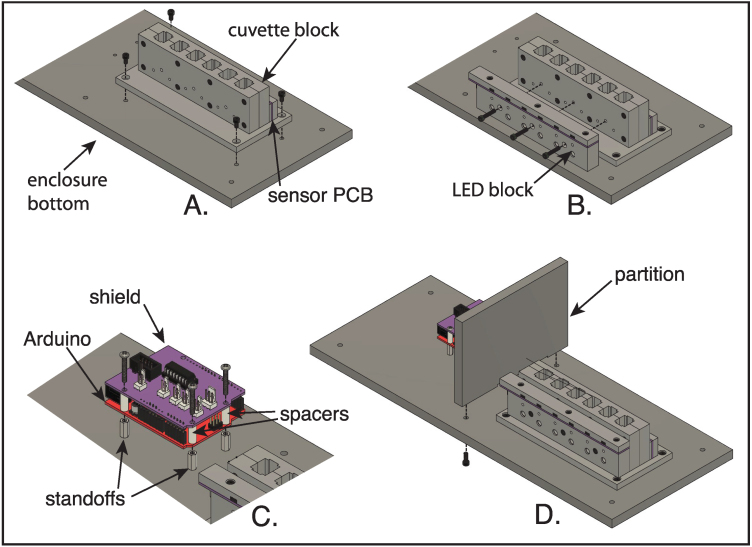
Assembly of major components to the enclosure bottom panel. (A) Attachment of the cuvette block to the enclosure bottom. (B) Attachment of the LED block to the cuvette block. (C) Assembly of the Arduino and shield PCBs onto standoffs onto the enclosure bottom. (D) Attachment of the enclosure partition to the bottom. In D, for clarity, the cables connecting the two sides of the instrument are not shown, but these should be connected before the partition is attached, and the cables fit into the recesses at the bottom of the partition.

1. Orient the enclosure bottom panel as shown in Fig. 21, with the side with the threaded inserts facing upwards and side with the counterbores facing downward. As shown in the figure, the holes with the 4–40 inserts should be on the left and the 6–32 inserts on the right.
2. Using four 6-32×12 in hex socket-head screws, attach the cuvette block assembly, with its baseplate and the sensors, to the enclosure bottom, as illustrated in Fig. 21A. These screws thread into the inserts in the matching holes at the left end of the enclosure bottom. The side of the block with sensors should face the back.
3. Attached the LED block, with the already attached circuit board and cover, to the cuvette block, using three 4-40×58 in hex socket-head screws (Fig. 21B).
4. Thread the three aluminum standoffs (4–40 × 716 in) into the three matching inserts on the left side of the enclosure bottom panel (Fig. 21C.).
5. If not already assembled, fit the Arduino shield to the Arduino board, as illustrated in Fig. 13, making sure to properly align the male and female headers.
6. Using three 4-40×34 in pan-head screws, attach the Arduino and shield boards to the standoffs, with 716 in long nylon spacers separating the two boards (Fig. 21C.).
7. Using the 2-conductor cable prepared in Section 5.3.2, connect the LED connector board (PC3) to the connector labeled “LEDs” on the Arduino shield.
8. Connect the shielded headers on the sensor board (PC2) and the Arduino shield, using the 8-conductor cable prepared in Section 5.3.1. Make sure that there are no twists in the cable.
9. Attach the partition between the cuvette block and Arduino boards to the enclosure bottom, using two 6-32×38 in round-head screws, inserted from below the bottom panel (Fig. 21D.). Note that the bottom of the partition has two shallow recesses to allow the cables between the cuvette block assembly and the Arduino shield to pass. The partition should be oriented with the narrower recess towards the front of the instrument, for the cable to the LEDs, and the wider recess towards the rear, for the 8-conductor cable to the sensor board. Carefully fit the two cables in these recesses before tightening the screws and ensure that the cables are not twisted or pinched.
10. Attach the rear aluminum panel (with the power switch and USB port) to the opening in the enclosure back, using four 6-32×38 in oval-head screws. The panel should be oriented with the USB port closest to the left end of the enclosure (as viewed from the front of the enclosure.)
11. With the enclosure bottom oriented as shown in Fig. 21D, lay the enclosure on its back behind the bottom panel. Connect the USB cable to the port on the Arduino board.
12. Connect the two conductor cable from the buzzer (previously mounted in the enclosure) to the Molex connector labeled ‘Tone’ on the Arduino shield.
13. Lower the enclosure over the bottom panel, being careful to keep any of the cables from being pinched.
14. Orient the aluminum panel above the opening in the enclosure top and, reaching into the enclosure, attach the cables from the panel to the Arduino shield, as described in Section 5.4.1. Lay the aluminum panel into its cutout in the top of the enclosure.
15. Before preceding to attach the aluminum panel to the enclosure top with screws, test the circuits again, as described in Section 5.4.3. For this test mount LEDs to each of the cuvette positions, using either six single-LED holders or a single holder with six LEDs. With the instrument fully assembled, absorbance readings should be very stable (fluctuating only in the third decimal place) and should correctly zero. If the circuits do not function correctly, the problem is most likely with one or more of the cable connections.
16. After confirming that the instrument is working correctly, attach the top panel to the enclosure with four 6-32×38 in oval-head screws.
17. Insert four 8-32×58 in round-head screws into the four rubber feet, inserting the screws into the sides of the feet with the larger opening. Then, use the screws to attach the bottom panel to the enclosure.
18. Attach the hinged cuvette-compartment door to the enclosure, following the steps below:
  (a) Cut a 6 in length of the piano hinge specified in the bill of materials. Each side of the hinge has pre-drilled holes separated by 2 in (50.8 mm), and the hinge should be cut so that each end extends by 1 in (25.4 mm) from the nearest hole.
  (b) Using three 4-40×14 in button-head screws, attach the hinge to the door assembly.
  (c) Place the door in position over the cuvette chamber and attach the hinge to the enclosure body, using three 4-40×14 in button-head screws.

## 6 Operation instructions

### 6.1 Preparation for use

**Fig. 22:**
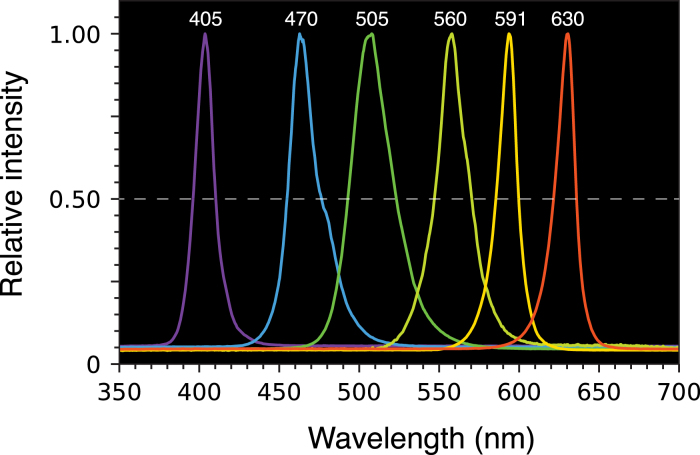
Emission spectra for six LEDs listed in the Bill of Materials, identified by the wavelength of maximum emission as reported by the manufacturers. The spectra were recorded using an Ocean Optics USB4000 VIS-NIR-ES spectrometer and have been normalized to a common maximum intensity. The widths of the spectra (FWHM) were estimated by fitting the data to a Gaussian function and were as follows for the six LEDs: 15 nm (405 nm), 25 nm (470 nm), 32 nm (505 nm), 27 nm (560 nm), 16 nm (591 nm), 16 nm 630 nm.

**Fig. 23:**
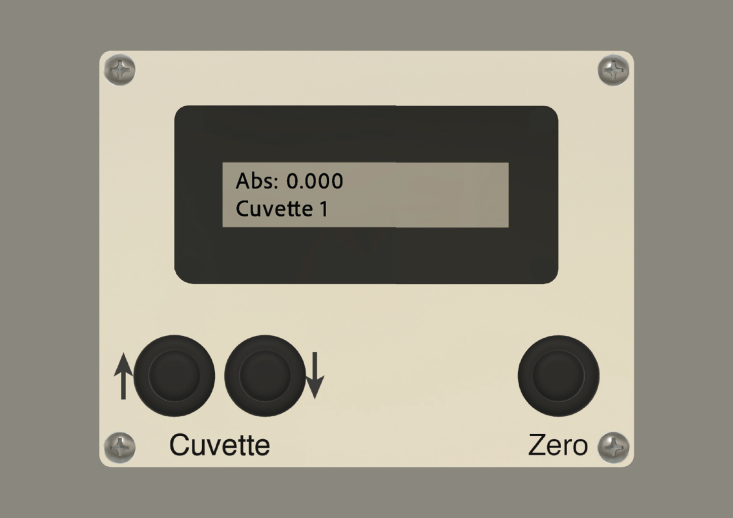
Control/display panel as it should appear after the spectrophotometer is turned on.

1. LED and dropping-resistor choice. The major preliminary step to using the LED spectrophotometer is choosing and installing an appropriate LED for the application. For optimum results, the emission spectrum should match as closely as possible the absorption spectrum of the compound of interest. The spectra for six LEDs listed in the Bill of Materials, with wavelengths of maximum intensity from 402 to 630 nm, are shown in Fig. 22. As listed in the figure legend, the width of the spectra ranged from 16 to 32 nm. Because absorption spectra are typically this wide or wider, the width of the LED spectra will not greatly affect most absorbance measurements, though it does affect the linearity of the response to concentration, as discussed in Sections 7.1–7.2. In addition, the instrument is unlikely to be suitable for experiments based on small spectral shifts.
  Although LEDs can be obtained from many sources, it is strongly recommended that they be purchased from vendors (such as Mouser and Digi-Key) that provide manufacturer’s datasheets. These datasheets will typically include emission spectra and specifications for luminous intensity and electrical parameters. In general, more narrow spectra will lead to better results, and it is particularly important to avoid LEDs with more than one emission peak (as found with some colored LEDs sold for consumer applications).
  The brightness of LEDs are usually reported as luminous intensity ($I_{v}$), with units of candela (cd), at a specified current (typically 20 mA for 5 mm LEDs of the type used here). For many wavelengths in the visible range, LEDs are available with $I_{v}$ of to 5 to 30 cd, and intensities in this range are well matched to the TSL235R sensor. An exception appears to be the range of approximately 560 to 570 nm, for which the luminous intensities of readily available LEDs are approximately 500-fold smaller, but still within the measurement range of the sensor.
  Because the voltage drop across an LED (like other diodes) is nearly constant, and these devices are very sensitive to excess current, a means of limiting the voltage and current is essential. (See https://en.wikipedia.org/wiki/LED_circuit.) In the instrument described here, this requirement is met by the simple expedient of placing a voltage-dropping resistor for each LED in the LED modules (see Sections 5.2.4, 5.2.5). For LEDs of the type used here and a supply voltage of 5 [V], a resistance of 330 Ω results in a current of about 10 mA, well below the typical maximum current of about 20 mA for 5 mm LEDs.
  For some LEDs, however, it may be necessary to use a larger resistance in order to limit the light output. The TSL235R sensor produces a square-wave output signal with a frequency that is proportional to the light intensity, up to a frequency of 500 kHz. Above this frequency, the sensor becomes saturated, leading to a loss of linearity in the output. The frequency output of the sensor can be easily determined by holding down the Zero and Cuvette increment (⇑) buttons of the spectrophotometer. Of the LEDs listed in the Bill of Materials, only the BIVAR 405 nm LED was found to saturate the sensor using a 330 Ω resistor. For this LED, a resistance of 660 Ω was found to result in an appropriate signal.
2. Cuvettes. The spectrophotometer is designed to use common cuvettes with a 1 cm × 1 cm total cross-section. Either standard (∼3 mL) or semi-micro (∼1 mL) cuvettes can be used. Both types of cuvettes have the same light-path length (1 cm) and outside dimensions. Although glass or fused-silica cuvettes can be used, much-less expensive plastic cuvettes are entirely satisfactory for the visible wavelengths used by this instrument.
3. Preparing the spectrophotometer. Before use, the appropriate LED modules should be installed and fastened to the LED block using the nylon thumbscrew. The modules for a single LED include a two-pin connector that fits into a matching connector at the top of the LED block, and the LED fits into a matching opening in the block, below the hole for the thumbscrew. The modules for six LEDs have only a single two-pin connector, which fits into the connector on the left side of the LED block, and two thumbscrews.
  The spectrophotometer is powered via the USB B connector at the back of the instrument, using a cable with a USB B connector at one end and a USB A connector at the other. The instrument requires only 0.4 W and can easily be powered by a mobile phone charger or computer USB port. Beware, however, that some portable, battery-powered USB power supplies (power banks) may not be suitable for powering the spectrophotometer, because the instrument may not draw the minimum current required to keep these supplies on.
4. Turn on the instrument using the power switch on the back panel. The instrument will go through a short warm-up period that includes setting the absorbance reading for each cuvette position to zero, though the displayed value will likely drift slightly as the instrument warms up. The control/display panel should appear as shown in Fig. 23
  The LCD display shows two lines, which usually indicate the current absorbance reading and the cuvette position being read.

### 6.2 Direct control of the instrument

The LED spectrophotometer has only three buttons to control its functions, as shown in Fig. 23 and described below:

• Changing the read position. The instrument will initially be set to read from cuvette position 1. The reading position can be changed by pressing either the cuvette increment (⇑) or decrement (⇓) button. Pressing both buttons simultaneously will set the read position to 1.
• Zeroing the read position. To set the absorbance reading to zero, press the Zero button. The display will briefly show a message indicating that zeroing is in progress, and then will return to the absorbance display. Note that each reading position must be zeroed individually. If the Zero and ⇓ buttons are pressed simultaneously, the instrument will cycle through all of the read positions and zero each of them.
• Reading light intensity. To read the unconverted light intensity recorded by the active sensor, simultaneously press the Zero and ⇑ buttons. The value shown in the top line of the display is the frequency of the signal output by the TSL235R sensor. This function is useful for testing whether or not the output of an LED lies within the linear response range of the sensor. If the value displayed is greater than 500,000, a larger dropping resistor should be used in the LED module

### 6.3 Spectrophotometer control via the USB port

The Arduino board of the spectrophotometer can receive and send text data via a serial interface to the USB port. With a suitable program, a computer connected to the spectrophotometer can be used to control the instrument and collect data. The computer software should be configured with the following parameters for the serial connection: baud = 9600, data bits = 8, parity bits = none, stop bits =1. The Arduino sketch, LEDSpec.ino, accepts the following commands via the USB port:

• check: Returns the string “imhere”. Used to check that the connection and instrument are working properly.
• cuvset: Followed by an integer in the range 1–6. Sets the active cuvette position. Values other than 1–6 are ignored.
• readabs: Returns the absorbance reading of the active cuvette position.
• readi: Returns the unprocessed frequency of the signal from the TSL235R sensor, an integer value that is proportional to the light intensity.
• zero: Sets the absorbance reading to zero for the active cuvette position.
• zeroall: Steps through all six cuvette positions and sets the absorbance reading of each to zero.
• beep: Generates a tone through the buzzer.
• beeepon: Turns the beep function on or off. If the command is followed by the string “off”, the function is turned off. If the command is followed by any other string, or no string, the function is turned on.

Each command must be followed by the ASCII new line character (decimal: 10 or hexadecimal: 0 A). After the command has been executed by the spectrophotometer, and the instrument is ready to receive another command, the string “ready” is returned to the connected computer. (An exception is the check command, which returns the string “imhere”.) If the instrument receives a command that is not recognized, it returns the string, “What?”.

A program for MacOS that utilizes this command set, MacSpec, is available from the author. This program is primarily designed to control the spectrophotometer and collect data for kinetic experiments.

### 6.4 Absorbance measurements

Once the instrument is set up, making absorbance measurements is a simple two-step procedure, common to all instruments of this type.

1. Zeroing the spectrophotometer with a blank solution. The blank solution should ideally contain all of the components of the solution to be analyzed except the compound of interest. Place a sample of the blank solution in a clean cuvette and then place the cuvette in the current read position. If using a semi-micro cuvette, the long path-length of the cuvette should be aligned with the light path, that is from front to back of the instrument. Press the Zero button. If additional cuvette positions are to be used for the same experiment, move the cuvette to each position, change the read position with the ⇑ or ⇓ buttons, and zero each position.
2. Read the absorbance of a sample. Remove the blank solution from the cuvette and replace it with the sample to measured. Place the cuvette in the appropriate position of the spectrophotometer and then wait a few seconds for the absorbance reading to stabilize before recording the value.

## 7 Validation and characterization

### 7.1 Absorbance measurements of *p*-nitroaniline

**Fig. 24:**
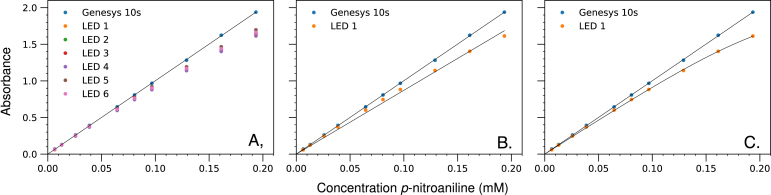
Absorbance as a function of *p*-nitroaniline concentration, as measured by a Thermo Fisher Scientific Genesys 10s UV–Vis spectrophotometer and the LED spectrophotometer. The wavelength of the Genesys 10s spectrophotometer was set to 405 nm, and the LED spectrophotometer was used with six different LEDs, each with nominal wavelength of 405 nm, fitted to the six positions of the instrument. The Genesys 10s instrument was used to determine the concentration of the stock solution of *p*-nitroaniline used for the measurements. In panel A, the data from the Genesys 10s and all six LEDs are plotted, and the results from the commercial instrument are fit to the Beer–Lambert law (Eq. (3)). In panels B and C, the data from only one of the LEDs are shown for clarity. In panel B, the data from the LED spectrophotometer are fit to the Beer–Lambert law, and in panel C, the same data are fit to the absorbance function defined by Eq. (7). The fit parameters for all of the measurements are listed in Table 1.

The measurement of solute concentrations by absorbance spectrophotometry rests on the Beer–Lambert law, which states that the absorbance (as defined by Eq. (1)) due to a compound is proportional to the solute concentration according to the relationship:

(3) A=Clϵ

where C is the concentration of the compound of interest, l is the cuvette path length (1 cm here) and ϵ is the extinction coefficient, which is a property of the compound and depends on the wavelength of light used and solution conditions. To examine the response of the LED spectrophotometer to concentration, absorbance measurements were made using solutions of *p*-nitroaniline ranging in concentration from 0.0065 to 0.19 mM. *p*-nitroaniline absorbs strongly at 405 nm, with an extinction coefficient at that wavelength of 1 × 10^4^ M^-1^cm^-1^[29]. This compound is the product of the enzymatic hydrolysis of synthetic substrates widely used to measure the activities of proteases (as illustrated in Section 7.2). The results of these measurements are graphed in Fig. 24

For this experiment, six different 405 nm LEDs were used in the six positions of the LED spectrophotometer, and the results from the six positions are plotted in panel A of Fig. 24, along with the results obtained with a commercial spectrophotometer, a Thermo Fisher Scientific model Genesys 10s uv-vis. The data from the Genesys 10s were fit to the Beer–Lambert law (with l fixed at a value of 1 cm). As shown, this instrument displays excellent linearity with concentration up to an absorbance value of at least 2.0. The fit value of the extinction coefficient was 1.0 × 10^4^ M^-1^cm^-1^, as expected since this value was used, with the same instrument, to determine the concentration of the stock *p*-nitroaniline solution.

As shown in Fig. 24A, the absorbance values determined using the LED spectrophotometer were systematically lower than those obtained using the commercial instrument, and this difference became more pronounced at higher solute concentrations. This result is not unexpected since the LEDs emission spectrum is substantially broader than that produced by a monochromator, and a fraction of the LED light is expected to be absorbed less efficiently. The six LEDs also displayed substantial differences in the degree to which the absorbance curves departed from that of the commercial instrument, suggesting that there is some variation in the emission spectra of different LEDs. In panel B of Fig. 24, the data for one of the LEDs, for which the absorbance values were lowest, are plotted along with the data for the commercial spectrophotometer, along with lines representing fits of the data sets to the Beer–Lambert law. For this LED, the estimated extinction coefficient was 8.7 × 10^3^ M^-1^cm^-1^. For the six LEDs, the apparent extinction coefficients ranged from 8.7 × 10^3^ to 9.1 × 10^3^ M^-1^cm^-1^ and are listed in the second column of Table 1.

Although the data from the LED spectrophotometer were well fit using the simple Beer–Lambert law, the data displayed a distinct downward curvature, as would be expected if there was a fraction of incident light that is less efficiently absorbed and becomes more significant as the total absorbance increases. A simplified model to account for this effect can be derived by assuming that there are two components of the light produced by the LED, one of which is absorbed according to the Beer–Lambert law, and one that is not absorbed at all. If the fraction that is not absorbed is defined as $f_{\mathrm{na}}$, the intensity of non-absorbable light entering and passing through the cuvette can be represented as:

(4) $$I_{\mathrm{na}}=I_{0}f_{\mathrm{na}}$$

and the initial intensity of absorbable light is:

(5) $$I_{\mathrm{0,a}}=I_{0}\left(1-f_{\mathrm{na}}\right)$$

From the Beer–Lambert law, the intensity of absorbable light that passes through the cuvette is:

(6) $$I_{a}=I_{0}\left(1-f_{\mathrm{na}}\right)10^{-Cl\epsilon }$$

The apparent absorption of light, as measured by the LED spectrophotometer, is then :

(7) $$A_{\mathrm{app}}=\log\frac{I_{0}}{I_{a}+I_{\mathrm{na}}}=-\log\left(\left(1-f_{\mathrm{na}}\right)10^{-Cl\epsilon }+f_{\mathrm{na}}\right)$$

**Table 1:** Parameters derived from fitting absorbance versus concentration data (Fig. 24) to the Beer–Lambert law (Eq. (3)) and to Eq. (7). Least-squares fits were performed using the curve_fit function of the SciPy Python package, and the uncertainties shown are derived from the fits. The coefficient of determination ($R^{2}$) was greater than 0.99 for all of the fits.

Spectrophotometer	Fit to Beer–Lambert law	Fit to Eq. (7)	
	$\epsilon \times 10^{-3}$ (M${}^{-1}$cm${}^{-1}$)	$\epsilon \times 10^{-3}$ (M${}^{-1}$cm${}^{-1}$)	$f_{\mathrm{na}}$
Genesys 10s	10.0±0.016	10.0±0.034	0.0000±0.0003
LED 1	8.71±0.11	9.43±0.05	0.0097±0.0006
LED 2	8.93±0.11	9.60±0.06	0.0083±0.0006
LED 3	8.87±0.11	9.56±0.05	0.0088±0.0006
LED 4	8.71±0.11	9.39±0.05	0.0092±0.0006
LED 5	9.11±0.11	9.76±0.06	0.0075±0.0006
LED 6	8.94±0.11	9.61±0.06	0.0083±0.0006

This function was fit to each of the data sets plotted in Fig. 24. The fits to the data from the Genesys 10s spectrophotometer and one of the LEDs is plotted in panel C of the figure, and the fit parameters for all of the LEDs are listed in Table 1. As shown in the example plotted in Fig. 24C, the data were very well fit by the function, which reproduced the observed curvature. For the Genesys 10s spectrophotometer, the fit value of $f_{\mathrm{na}}$ was zero, as expected, and the fit value of the extinction coefficient was the same as derived from the Beer–Lambert law. For the six LEDs, the fit values of $f_{\mathrm{na}}$ ranged from 0.0075 to 0.0097, indicating that only about 1% of the light from the LEDs is not absorbable. This fraction has a relatively small impact when the total absorption is less than about 1, but becomes more significant at higher absorbance values.

In most practical applications, the deviation from the Beer–Lambert law can be effectively minimized by limiting absorbance measurements to less than about 1. Even under these conditions, however, the apparent extinction coefficient should be determined by measuring absorbance as a function of concentration of the compound of interest, using samples with independently determined concentrations.

### 7.2 Measurement of the kinetics of an enzymatic reaction

To demonstrate the suitability of the instrument for studies of chemical and biochemical reaction kinetics, it was used to measure the rate of hydrolysis of a synthetic substrate by the enzyme trypsin. The substrate, benzoyl-phenylalanyl-valyl-arginine-4-nitroanilide (FVA-na) does not significantly absorb light with wavelengths in the range of 400 nm, but releases *p*-nitroaniline upon hydrolysis, allowing the reaction to be monitored spectrophotometrically [30]. For this experiment, six cuvettes, containing the same concentration of substrate and different concentrations of trypsin, were set up in the LED spectrophotometer (using the same set of LEDs as in the experiment described above) and the reactions were monitored simultaneously, over a period of 5 min using the MacSpec program to control the spectrophotometer and record absorbance readings at 0.5 min intervals.

**Fig. 25:**
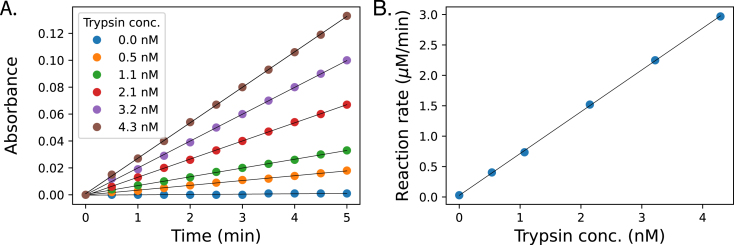
Measurement of enzyme kinetics using the LED spectrophotometer. The hydrolysis of benzoyl-phenylalanyl-valyl-arginine-4-nitroanilide by bovine β-trypsin was measured by monitoring the absorbance of light emitted by 405 nm LEDs, as used in the experiment illustrated in Fig. 24. The reactions were carried out in plastic semi-micro cuvettes containing a total reaction volume of 0.8 mL. The reaction solutions contained: 0.4 mM substrate; 0.1 M Tris-Cl buffer, pH 8; 2 mM CaCl_2_; and the indicated concentration of trypsin. In panel A, the absorbance values recorded from each reaction, after subtracting the value measured at time zero, are plotted as a function of time. In panel B, the rates of change in concentration are plotted as a function of enzyme concentration. The lines plotted in both panels represent linear least-squares fits to the data. The coefficients of determination ($R^{2}$) for all of the fits were greater than 0.99, except for the data in Panel A for the reaction with no trypsin, where the slope is nearly 0.

The resulting absorbance measurements are plotted as a function of time in Fig. 25A. The values plotted in the graph have been corrected by subtracting the values recorded at the first time points, so the rates of change in absorbance can be compared more easily than if the absolute absorbances were plotted. For each of the reactions, the absorbance increased linearly with time, as expected, because only a small fraction (4% or less) of the substrate was consumed during the reactions. The data were analyzed by fitting a straight-line function (y=mx+b) to estimate the rates of change in absorbance, from which rates of change of concentration were calculated from the Beer–Lambert law, using a value of 8.88 × 10^3^ cm^-1^M^-1^ for the extinction coefficient (the mean of values determined from the experiment shown in Fig. 24).

The calculated reaction rates are plotted as a function of enzyme concentration in panel B of Fig. 25. As predicted by the Michaelis–Menten equation for enzyme kinetics, the rates were proportional to enzyme concentration and were well fit by a linear function (y=mx+b) that passes very close to the origin (y-intercept = 0.0.026 μM/min).

These experiments demonstrate that the LED spectrophotometer is capable of producing data of very high quality, provided that its limitations are taken into account. Most importantly, for absolute measurements of concentration, apparent extinction coefficients should be determined experimentally, using a conventional spectrophotometer to establish the concentrations of standard solutions, and the range of linearity should be determined for each combination of solute compound and LED type.

### 7.3 Suggestions for further refinements

One important way in which the instrument described here could improved is by reducing its cost, much of which is due to the use of CNC-machined components. It should be possible to fabricate most, if not all, of the plastic components using 3-d printing technology, which may reduce their cost. All of the components have relatively simple shapes, and the STEP-format CAD files can be converted to formats suitable for 3d-printing. Printing components with the precision and fit obtained by machining will likely require the use of a material with a higher melting temperature than the polylactic acid (PLA) most commonly used with inexpensive table-top printers. For this purpose, acrylonitrile butadiene styrene (ABS) or nylon may be suitable.

A less expensive, and more compact, version of the instrument could also be built by simplifying the design so that there is only a single cuvette position. Although this would eliminate a unique features of the design, a simplified version would likely be suitable for many educational or field applications.

In the future, it may also be necessary to adapt the design to a different light sensor. As of July 2025, the TSL235R was not in production, but supplies were readily available. Possible replacements of the TSL235 for this application include the TSL2561 and TSL2591 (both manufactured by OSRAM), but using these devices would require significant redesign of the circuit board on which the detectors are mounted (PC2), the electronic circuit and some of the plastic components.

## Human and animal rights

The work described here did not involve the use of human subjects or animals.

## Declaration of competing interest

The author declares no competing interests.

## Footnotes

2 Sketches can also be loaded onto some Arduino and compatible boards using the Arduino Cloud web site (https://cloud.arduino.cc/). The SparkFun RedBoard used in our prototypes is not currently supported by this service, though the very similar Arduino Uno boards are supported. Other Uno-compatible boards may or may not be supported.

## Footnotes

1 The language used to describe instruments of this and related types can be confusing. The term spectrophotometer is also commonly used for instruments used for measuring the spectrum of light emitted from computer displays or reflected from printed material. The term spectrometer is generally used more broadly for instruments that measure the spectra of emission or absorbance of radiation, from a wide variety of sources. Absorbance instruments such as the one described here are also sometimes referred to as colorimeters, to indicate that they operate using a limited number of fixed wavelengths, as compared to the continuously adjustable light sources used in more expensive instruments.

## References

[1] Rouessac F., Rouessac A. (2007).

[2] Flaschka H., McKeithan C., Barnes R. (1973). Light emitting diodes and phototransistors in photometric modules. Anal. Lett..

[3] Hauser P.C., Rupasinghe T.W.T., Cates N.E. (1995). A multi-wavelength photometer based on light-emitting diodes. Talanta.

[4] Cantrell K.M., Ingle J.D. (2003). The SLIM spectrometer. Anal. Chem..

[5] Yeh T.-S., Tseng S.-S. (2006). A low cost LED based spectrometer. J. Chin. Chem. Soc..

[6] Yang H., Wei X., Liang X., Su M., Lu X. (2008). A SoC and LED based reconfigurable subminiature sectrometer for hand-held measurement applications. Measurement.

[7] Albert D.R., Todt M.A., Davis H.F. (2012). A low-cost quantitative absorption spectrophotometer. J. Chem. Educ..

[8] Butterfield A.E., Young C.C. (2012). An effective and economical photometer for classroom demonstrations and laboratory use. Chem. Eng. Educ..

[9] Anzalone G.C., Glover A.G., Pearce J.M. (2013). Open-source colorimeter. Sensors.

[10] Asheim J., Kvittingen E.V., Kvittingen L., Verley R. (2014). A simple, small-scale lego colorimeter with a light-emitting diode (LED) used as detector. J. Chem. Educ..

[11] Bueno D., Alonso G., Muũnoz R., Marty J.L. (2014). Low-cost and portable absorbance measuring system to carbamate and organophosphate pesticides. Sensors Actuators B: Chem..

[12] McClain R.L. (2014). Construction of a photometer as an instructional tool for electronics and instrumentation. J. Chem. Educ..

[13] Santos W.G., Cavaheiro E.T.G. (2015). Assemblng and using an LED-based detector to monitor absorbnce changes during acid–base transitions. J. Chem. Educ..

[14] Wang J.J., Nuñez J.R.R., Maxwell E.J., Algar W.R. (2015). Build your own photometer: A guided-inquiry to introduce analytical instrumentation. J. Chem. Educ..

[15] Bougot-Robin K., Paget J., Atkins S.C., Edel J. (2016). Optimization and design of an absorbance spectrophotometer controlled using a raspberry Pi to improve analytical skills. J. Chem. Educ..

[16] Clippard C.M., Hughes W., Chohan B.S., Sykes D.G. (2016). Construction and characterization of a compact portable, low-cost colorimeter for the chemistry lab. J. Chem. Educ..

[17] Porter L.A., Washer B.M., Hakim M.H., Dallinger R.F. (2016). User-friendly 3D printed colorimeter models for student exploration of instrument design and performance. J. Chem. Educ..

[18] Chaianantakul N., Wutthi K., Kamput N., Pramanpol N., Janphuang P., Pummara W., Phimon K., Phatthanakun R. (2018). Development of mini-spectrophotometer for determination of plasma glucose. Spectrochim. Acta Part A: Mol. Biomol. Spectrosc..

[19] Bogucki R., Greggila M., Mallory P., Feng J., Siman K., Khakipoor B., King H., Smith A.W. (2019). A 3-d printable dual beam spectrophotometer with multiplatform smartphone adaptor. J. Chem. Educ..

[20] O’Donoghue J. (2019). Simplified low-cost colorimetry for education and public engagement. J. Chem. Educ..

[21] Laganovska K., Zolotarjovs A., Vázquez M., McDonnell K., Liepens J., Ben-Yaav H., Karitans V., Smits K. (2020). Portable low-cost open-source wireless spectrophotometer for fast and reliable measurements. HardwareX.

[22] Siu V.S., Lu M., Hsieh K.Y., Raines K., Asaad Y.A., Pateel K., Afzali-Ardakani A., Wen B., Budd R. (2022). Toward a quantitative colorimeter for point-of-care nitrite detection. ACS Omega.

[23] Ondřej K., Miroslav P. (2023). Affordable portable platform for classic photometry and low-cost determination of cholinesterase activity. Biosensors.

[24] Kulkarni P.S., Watwe V.S., Nirmal R.S., Jagdale H.N., Kulkarmi S.D. (2024). Simultaneous determination of Ni(II) and Co(II) in aqueous solution using an image-based do-it-yourself photometer. J. Chem. Educ..

[25] Kovarik M.L., Clapis J.R., Romano-Pringle K.A. (2020). Review of student-built spectroscopy instrumentatin projects. J. Chem. Educ..

[26] (2016). https://www.youtube.com/watch?v=eUcdSSBDEEI.

[27] (2015). https://www.youtube.com/watch?v=8jcfD1UW8SE.

[28] Millman M. (2020). https://www.mattmillman.com/info/crimpconnectors/.

[29] Lottenberg R., Jackson C.M. (1983). Solution composition dependent variation in extinction coefficients for *p*-nitroaniline. Biochim. Biophys. Acta - Protein Struct. Mol. Enz..

[30] Svendsen L., Blombäck B., Blombäck M., Olsson P.I. (1972). Synthetic chromogenic substrates for determination of trypsin, thrombin and thrombin-like enzymes. Thromb. Res..

